# Endocrine Manifestations and New Developments in Mitochondrial Disease

**DOI:** 10.1210/endrev/bnab036

**Published:** 2021-10-13

**Authors:** Yi Shiau Ng, Albert Zishen Lim, Grigorios Panagiotou, Doug M Turnbull, Mark Walker

**Affiliations:** Wellcome Centre for Mitochondrial Research, Translational and Clinical Research Institute, Newcastle University, Newcastle upon Tyne, UK; NHS Highly Specialised Service for Rare Mitochondrial Disorders, Newcastle upon Tyne Hospitals NHS Foundation Trust, Newcastle upon Tyne, UK; Wellcome Centre for Mitochondrial Research, Translational and Clinical Research Institute, Newcastle University, Newcastle upon Tyne, UK; NHS Highly Specialised Service for Rare Mitochondrial Disorders, Newcastle upon Tyne Hospitals NHS Foundation Trust, Newcastle upon Tyne, UK; Department of Diabetes and Endocrinology, Newcastle upon Tyne Hospitals NHS Foundation Trust, Newcastle upon Tyne, UK; Wellcome Centre for Mitochondrial Research, Translational and Clinical Research Institute, Newcastle University, Newcastle upon Tyne, UK; NHS Highly Specialised Service for Rare Mitochondrial Disorders, Newcastle upon Tyne Hospitals NHS Foundation Trust, Newcastle upon Tyne, UK; Department of Diabetes and Endocrinology, Newcastle upon Tyne Hospitals NHS Foundation Trust, Newcastle upon Tyne, UK; Translational and Clinical Research Institute, Newcastle University, Newcastle upon Tyne, UK

**Keywords:** mitochondrial DNA, diabetes mellitus, MIDD, clinical management, genomic testing, reproductive options

## Abstract

Mitochondrial diseases are a group of common inherited diseases causing disruption of oxidative phosphorylation. Some patients with mitochondrial disease have endocrine manifestations, with diabetes mellitus being predominant but also include hypogonadism, hypoadrenalism, and hypoparathyroidism. There have been major developments in mitochondrial disease over the past decade that have major implications for all patients. The collection of large cohorts of patients has better defined the phenotype of mitochondrial diseases and the majority of patients with endocrine abnormalities have involvement of several other systems. This means that patients with mitochondrial disease and endocrine manifestations need specialist follow-up because some of the other manifestations, such as stroke-like episodes and cardiomyopathy, are potentially life threatening. Also, the development and follow-up of large cohorts of patients means that there are clinical guidelines for the management of patients with mitochondrial disease. There is also considerable research activity to identify novel therapies for the treatment of mitochondrial disease. The revolution in genetics, with the introduction of next-generation sequencing, has made genetic testing more available and establishing a precise genetic diagnosis is important because it will affect the risk for involvement for different organ systems. Establishing a genetic diagnosis is also crucial because important reproductive options have been developed that will prevent the transmission of mitochondrial disease because of mitochondrial DNA variants to the next generation.

## Graphical Abstract

**Graphical Abstract:**
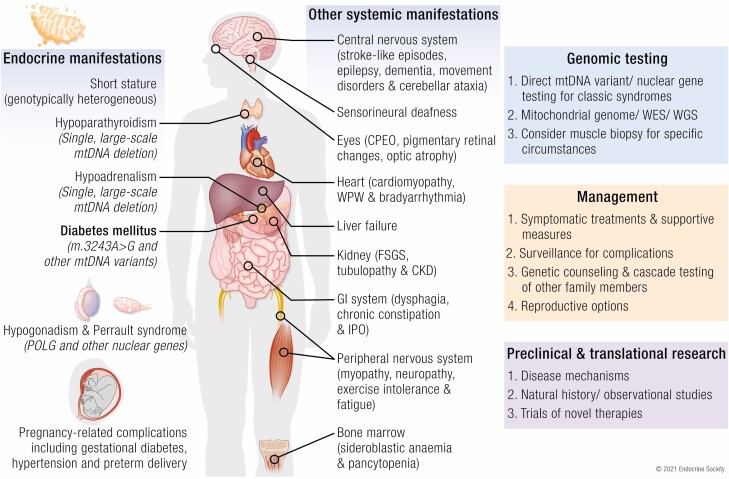

**Essential Points**

- The most frequent endocrine problem in adult patients with mitochondrial disease is diabetes mellitus and that this is largely because of the common m.3243A > G mtDNA pathogenic variant. This form of diabetes mellitus is often accompanied by sensorineural deafness.
- Patients with single, large-scale mtDNA deletions may develop several different endocrine phenotypes and that patients with childhood-onset severe disease should be closely monitored.
- Involvement of different nuclear genes encoding important mitochondrial proteins may lead to very specific endocrine problems—for example, primary ovarian failure in *POLG* disease.
- The investigation of mitochondrial disease has been simplified with the development of next-generation sequencing and identifying a genetic diagnosis crucial for management, providing appropriate genetic advice and enrolment to clinical trials.
- For most patients with mitochondrial disease, there are no specific curative treatments currently and management of endocrine problems is similar to other patients with hormone deficiency.
- It is crucial that patients with endocrine dysfunction from mitochondrial disease are carefully monitored for other complications of mitochondrial disease—for example, the cardiac or gastrointestinal symptoms seen in patients with m.3243A > G disease.
- There are new reproductive options for patients with inherited mitochondrial DNA pathogenic variants; these options should be discussed early with patients and families.

Mitochondria play an important role in cellular function in all tissues, including those of the endocrine system. Indeed, endocrine defects such as diabetes mellitus are prevalent in certain forms of mitochondrial disease. Over the past decade, there have been major advances in our knowledge of mitochondrial disease, including advances in diagnosis, management, and prevention (1). This review will focus specifically on the role of mitochondrial abnormalities causing endocrine problems, new developments in diagnosis, emerging therapies and reproductive options, and how these advances in our understanding of mitochondrial disease are influencing patient management.

## Background Nature of Mitochondrial Biology and Genetics

**Figure 1.**
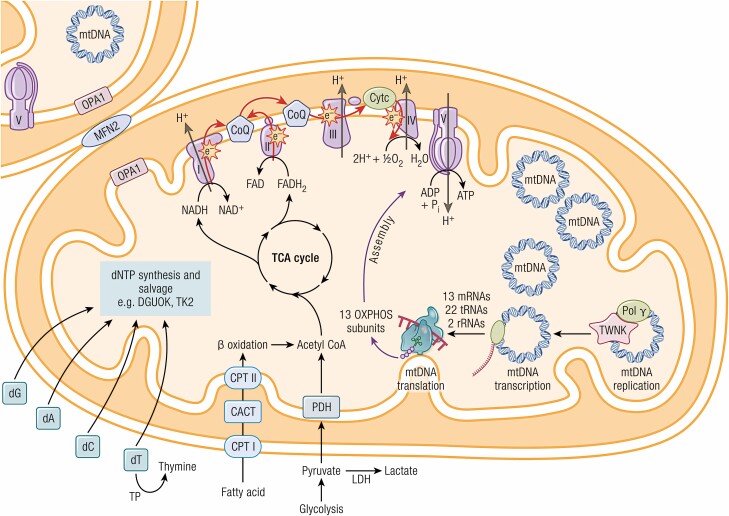
Mitochondrial oxidative phosphorylation (OXPHOS) system and other pathways that are commonly implicated in mitochondrial diseases. The OXPHOS system comprises complexes I to V and 2 mobile electron carriers, CoQ_10_ and cytochrome *c*. The breakdown of carbohydrate (glycolysis) and fatty acids (beta oxidation) lead to the production of acetyl-coenzyme A (CoA), which is the first substrate of the TCA cycle (also known as the citric acid cycle or Krebs cycle). NADH and FADH_2_ are generated through a series of enzymatic reactions in which electrons are transferred along the mitochondrial respiratory chain (complex I-IV). High-energy electrons are passed along the complexes and protons (H^+^) are pumped out of the matrix space, creating an electrochemical membrane potential that is used by the ATP synthase (complex V) to phosphorylate ADP and generate ATP. The mtDNA encodes 13 protein subunits, 22 tRNAs and 2 rRNAs; there are multiple copies of mtDNA per cell, ranging from hundreds to thousands depending on the cell type. The replication, maintenance, transcription, and translation of mtDNA and mtDNA-encoded proteins are dependent on many nuclear-encoded proteins that are synthesized in the cytosol and imported into mitochondria through specific transporters (not shown). Genetic defects in nucleotide synthesis and salvage (eg, *DGUOK*, *TYMP*, *TK2*), mtDNA replication and maintenance (eg, catalytic subunit [*POLG*] and accessory units [*POLG2*] of polymerase gamma and *TWNK*), fusion and fission machinery (eg, *MFN2*, *OPA1*) can perturb mtDNA integrity and copy number, leading to the formation of multiple deletions and mtDNA depletion, respectively. This figure is derived from a previous published work (1).

Mitochondria are ubiquitous organelles present in all nucleated cells in the body. Mitochondria have multiple functions within cells, including oxidative phosphorylation, fatty acid oxidation, Krebs cycle, urea cycle, gluconeogenesis, and ketogenesis (**Fig. 1**). They also play an important role in several other important cellular processes including (nonshivering) thermogenesis, amino acids and lipid metabolism, biosynthesis of haem and iron-sulphur clusters, calcium homeostasis, and apoptosis. Mitochondria are dynamic organelles undergoing fission and fusion depending on the metabolic state of the cell (2).

Oxidative phosphorylation (OXPHOS) occurs in the inner mitochondrial membrane, and there are 5 multisubunit complexes that are directly involved in OXPHOS, 3 of which pump protons into the intermembrane space (complexes I, III, and IV). This generates an electrochemical gradient across the inner membrane that dissipates through complex V to generate ATP from ADP and Pi (**Fig. 1**). The complexes involved in OXPHOS include subunits encoded by both mitochondrial and nuclear genomes highlighting the complex genetics of mitochondria.

The mitochondrial genome is a small (16.6 kb) circular DNA (mtDNA) that contains 37 genes, 13 protein subunits of OXPHOS, 22tRNAs, and 2 rRNAs. The 13 protein subunits consist of 7 subunits of complex I, 1 subunit of complex III, 3 subunits of complex IV, and 2 subunits of complex V. The rRNA molecules enable the intramitochondrial synthesis of these subunits. The rest of the mitochondrial proteins, including all the other subunits of OXPHOS, are nuclear encoded and transferred into mitochondria after synthesis in the cytosol.

In view of the multiple functions of mitochondria, the diseases that are traditionally (and in this review) termed mitochondrial are those in which defects of OXPHOS are the primary abnormality. This therefore includes those genetic defects involving the mitochondrial genome and specific nuclear genes involved directly or indirectly in OXPHOS. There are approximately 1100 nuclear mitochondrial proteins, including those important for the many other functions of mitochondria (3). Nuclear mitochondrial proteins are also directly, and indirectly, involved in the replication and repair of mtDNA, and thus nuclear mitochondrial defects can lead to depletion or pathogenic variants of the mitochondrial genome (4). Currently, mitochondrial disease genes can be classified based on their functional roles and pathways involved (3, 5-7): (1) OXPHOS structural subunits, assembly factors and electron carriers; (2) metabolism of cofactors and vitamin; (3) metabolism of substrates including TCA cycle and fatty oxidation; (4) mtDNA replication, maintenance, transcription, RNA processing/maturation, and translation; (5) mitochondrial membrane dynamics, composition, and quality control; and (6) others (including proteins with functions that are less well-characterized). Clinically, defects of mtDNA and nuclear DNA can look very similar, which is why establishing a genetic diagnosis is crucial.

Mitochondrial DNA genetics is very different from nuclear DNA genetics (8). Mitochondrial DNA is maternally inherited and mtDNA diseases are only passed down the female line (9-11). Cells contain multiple copies of mtDNA ranging from several hundred in some cells to hundreds of thousands in an oocyte. In the presence of a pathogenic variant of the mitochondrial genome, this can affect all copies (called homoplasmy) or a mixture of wild-type and mutated copies (called heteroplasmy). Homoplasmic variants are an important cause of disease with well-recognized pathogenic variants, but the majority of pathogenic variants are heteroplasmic (12). Most heteroplasmic mtDNA defects are functionally recessive and thus high levels of mutated mtDNA are required before a biochemical defect in cells and the development of clinical disease. This threshold varies for different variants and indeed there may be a difference in clinical threshold for disease dependent on nuclear (13) or environmental factors (14, 15). From a clinical perspective, it is important to recognize there is often a difference between the level of heteroplasmy in different tissues with lower levels in replicating tissues (such as blood) and much higher levels in nondividing tissues (such as muscle) (12). This is important to recognize in the diagnosis of mtDNA disease (see Mitochondrial DNA genomics).

## Prevalence of mitochondrial disease

Mitochondrial diseases are one of the most commonly inherited metabolic disorders, with a prevalence in the adult population of approximately 1 in 5000 of the population. Epidemiological studies have shown that approximately two-thirds of all mitochondrial disease in adults is due to mtDNA pathogenic variants (16). The prevalence of all forms of childhood-onset (<16 years of age) mitochondrial diseases has been estimated to range from 5 to 15 cases per 100 000 individuals and to be predominantly the result of pathogenic variants in nuclear genes (approximately 80%). There is considerable variation in prevalence of childhood-onset mitochondrial disease in different populations because of genetic founder variants and high consanguinity (17). Pathogenic variants in more than 300 nuclear genes cause mitochondrial disease (5), some of which are associated with endocrine abnormalities.

## Clinical Features of Mitochondrial Disease—Endocrine

Disorders of the endocrine system are frequently reported in patients with specific subtypes of mitochondrial disease and defects of OXPHOS. Although diabetes is the predominant endocrine manifestation, abnormalities of other endocrine glands are also observed. Hormone deficiency (as opposed to secretory excess) is a consistent feature of mitochondrial endocrinopathies. This is not surprising because, in general, hormone synthesis and secretion are energy-dependent processes. In theory, therefore, all endocrine organs are prone to mitochondrial dysfunction. However, certain endocrine tissues seem to be particularly susceptible to mitochondrial dysfunction, leading to an increased prevalence of specific hormone deficiencies.

### Diabetes mellitus

Original reports describing the role of mtDNA variants in the development of human disease highlighted the impact on the neuromuscular system (18, 19). Two papers published in 1992 provided the first strong evidence of the association of diabetes mellitus with mitochondrial DNA pathogenic variants. The first described a pedigree in which there was maternal inheritance of diabetes mellitus and/or premature deafness across 3 generations because of a 10.4-kb mtDNA duplication/deletion (20). Of the 9 affected individuals, 7 had diabetes and deafness. All of the patients with diabetes were insulin treated, and 3 had experienced diabetic ketoacidosis. In the second report, 11 individuals within a large pedigree had non-insulin-dependent diabetes and deafness with a clear maternal pattern of inheritance. An A to G transition at position 3243 in the mitochondrial *MT-TL1* gene was identified that cosegregated with the diabetes and deafness (21, 22). From these initial landmark reports, it was apparent that the diabetes phenotype linked to mitochondrial dysfunction was variable and spanned insulin dependence through to non-insulin-dependent diabetes.

Subsequent papers followed, with the majority focusing on the m.3243A > G variant and its association with diabetes. Based on the expanding evidence base, Maassen and Kadowaki proposed the new diabetes subtype of maternally inherited diabetes and deafness (MIDD) because of the 3243 mtDNA variant (23); this subtype was later incorporated into the World Health Organization diabetes classification. Although other pathogenic mtDNA variants have been reported in associations with mitochondrial diabetes (see Prevalence of m.3243A > G MIDD and other mtDNA variants associated with diabetes mellitus), MIDD resulting from the m.3243A > G variant is the subtype that the endocrinologist is most likely to encounter in clinical practice and is the focus of this review.

#### Diabetes phenotype in MIDD

A key objective of this review is to provide guidance as to how to identify and investigate a patient with suspected MIDD. The accumulated evidence from case series and cohort studies has identified some common themes and highlighted areas of variation. The mean age of diagnosis of diabetes is remarkably similar between studies. In a multicenter study of 54 patients with MIDD and the m.3243A > G variant (24), the mean age of diabetes diagnosis was 39 years (range, 12-67 years). A review of 31 patients with MIDD attending a national mitochondrial disease service reported a mean age of diabetes diagnosis of 38 years for the same gene variant (25). Other studies have similarly reported that diabetes generally presents in early middle age, although with a broad age range (26). Although the mean age of diagnosis is consistent within m.3243A > G MIDD, age of diabetes diagnosis is of no real value in helping to discriminate from other forms of diabetes. That is because autoimmune diabetes (type 1 and late-onset diabetes of the adult) (27), and other monogenic forms of diabetes such as maturity-onset diabetes of the young (28, 29) can present in early middle age.

The diabetes phenotype is variable and dynamic. It was estimated that 13% of m.3243A > G MIDD patients required insulin therapy from the time of diagnosis (25), whereas 45% of the remaining non-insulin-dependent patients progressed to insulin therapy over a mean period of 4.2 years. In a cross-sectional review, 41% of patients with MIDD were classified as non-insulin dependent, whereas 13% required insulin from the time of diagnosis and 8% presented in diabetic ketoacidosis (30). Both studies were based in populations of North European extraction, but this variable diabetes phenotype is not restricted to this ethnic group. In studies of Japanese pedigrees, there was evidence of insulin and non-insulin-treated diabetes within pedigrees (26, 31, 32); in 1 family, 2 patients presented with diabetic ketoacidosis, whereas other relatives were non-insulin dependent (26). In essence, the diabetes phenotype is variable both within the MIDD subtype, and within affected pedigrees.

Despite this variable diabetes phenotype, it is important to note that m.3243A > G patients with MIDD are invariably lean at presentation (23). In a French cohort of 54 patients with MIDD, the mean body mass index (BMI) was 20.2 with a range of 13.5 to 27.1 kg/m^2^ (24). Whittaker and colleagues reported mean BMIs of 22.3 and 23.5 kg/m^2^, respectively, in diabetic and nondiabetic m.3243A > G variant carriers (25), whereas in a Japanese study the patients with MIDD had a mean BMI of 20.4 (±3.1 SD) kg/m^2^ (33). It is evident that irrespective of whether the patients with MIDD are insulin or non-insulin requiring, they are almost always nonobese (BMI < 30 kg/m^2^), which is an important consideration from a diagnostic perspective.

#### Lack of autoimmunity in MIDD

Autoimmune diabetes is characterized by elevated titers of specific autoantibodies, in particular glutamic acid decarboxylase (GAD) and islet tyrosine phosphatase 2 (34). The observation that some m.3243A > G patients with MIDD present with diabetic ketoacidosis and/or require insulin from the time of diagnosis raised the possibility of an autoimmune process in the development of diabetes. Suzuki and colleagues tested 78 patients with MIDD and none were autoantibody positive (33). Conversely, a study of 31 Japanese patients with MIDD identified 1 patient who was strongly positive for both GAD and islet cell antibodies (ICAs), likely to be coexisting autoimmune diabetes, whereas another 12 patients were weakly positive for ICAs (35). It was postulated that the low-grade ICA positivity in the absence of GAD antibodies in MIDD might represent an autoimmune response to partial beta-cell damage secondary to mitochondrial dysfunction (36). However, supporting mechanistic evidence is lacking, and a subsequent large multicenter study of 54 patients with MIDD identified ICA positivity in just 1 patient (24). As previously surmised, there is therefore no robust evidence that MIDD is an autoimmune-driven condition (37). However, clear GAD positivity is a marker of autoimmune diabetes that would, from time to time, be expected to coexist in families with MIDD.

#### Potential modulators of the MIDD diabetes phenotype

The variable diabetes phenotype in patients and their families with m.3243A > G MIDD results from the interplay of different factors. Among 5 members of the same family with m.3243A > G MIDD, the level of heteroplasmy in blood was found to range from 16% to 29% (38). Analysis of tissue from a postmortem donor with the m.3243A > G variant revealed that the level of heteroplasmy differed markedly between tissues, and varied between individual pancreatic islets (see Pathophysiology of mitochondrial diabetes). It is now recognized that many hundreds of common, but functionally weak, genetic susceptibility variants predispose to type 1 and type 2 diabetes (39). The clustering of such variants within families harboring the m.3243A > G variant would be predicted to modulate the diabetes phenotypes. First, family relatives who do not carry the m.3243A > G variant might develop classical type 1 or type 2 diabetes, which would confound the maternal inheritance pattern. Second, the diabetes phenotype may be modulated in relatives with the m.3243A > G variant by the clustering of type1 and/or type 2 diabetes variants. This has been recognized in maturity-onset diabetes of the young (40) and is likely to hold the same importance in MIDD.

#### Sensorineural deafness in MIDD

The m.3243A > G variant has been shown to cause symmetric sensorineural hearing impairment secondary to cochlear dysfunction (41, 42). The mean age of onset of hearing loss has been reported at 26 (43) and 34 (44) years. The hearing impairment starts with high frequency loss, and progresses steadily over time in the majority of patients (44). The level of m.3243A > G heteroplasmy was measured in tissues other than blood (almost exclusively muscle) from 40 individuals with the m.3243A > G variant, and was found to be higher in patients with hearing impairment compared with those matched for age with normal hearing (44).

Sensorineural hearing loss is a key feature of patients with mitochondrial diabetes resulting from the m.3243A > G, with a prevalence of clinically apparent hearing loss of between 86% and 98% in cross-sectional studies (24, 33). We found that hearing loss preceded the diagnosis of diabetes by an average of 6 years (25). This pattern also applied to patients with diabetes and deafness from other mitochondrial variants (25). Furthermore, patients with maternally inherited diabetes alone, without coexisting deafness and/or neuromuscular dysfunction, did not harbor the m.3243A > G variant or any other pathological variants on sequencing the entire mitochondrial genome (45).

Taking these observations together, a history of bilateral sensorineural hearing loss developing in early adulthood and before the onset of diabetes is a strong signal to investigate for MIDD.

#### Prevalence of m.3243A > G MIDD and other mtDNA variants associated with diabetes mellitus

An extensive and detailed review of the prevalence of MIDD resulting from the m.3243A > G variant was conducted by Murphy and colleagues (41). The summary findings were that the prevalence rates of MIDD ranged between 0.8% and 1.5% on average for unselected European and Japanese diabetic populations, respectively, increasing to an average of 5% when the screening was restricted to patients with diabetes and a personal and/or family history of deafness.

The evidence that the m.3243A > G variant causes diabetes came from studies of pedigrees that revealed cosegregation of the variant with diabetes, and the report of the high prevalence of diabetes (~40%) in patients with the variant attending a national mitochondrial clinical service (25, 46). This prevalence rate was much higher than the prevalence of the classical forms of diabetes mellitus in the background population. Diabetes mellitus has also been identified in patients with other mtDNA variants attending the same clinical service (25). The proportion (and percentage) of patients with diabetes with the following variants was: m.14709T > C: 7/13 (54%), single, large-scale mtDNA deletion: 6/55 (11%), m.8344A > G: 3/29 (10%) and multiple mtDNA deletions: 3/43 (7%). However, this was a cross-sectional analysis with no age adjustment. Using a different approach, the same group identified 29 patients who had presented with the classical MIDD phenotype, of which 21 were carriers of the m.3243A > G variant. The remaining 8 patients carried mitochondrial point variants (m.8344A > G and m.12258C > A) or single, large-scale mtDNA deletions. In all but one, deafness preceded the diagnosis of diabetes, and subsequent targeted clinical review revealed that 6 of these patients exhibited other features of mitochondrial disease (47). Taking this information together, it is evident that other mtDNA variants are associated with diabetes and can present as the MIDD phenotype, but the m.3243A > G is the predominant genotype.

As previously reviewed (48, 49), diabetes has been reported in patients who harbor mutations in nuclear-encoded genes involved with mtDNA maintenance. However, it can be difficult to discern whether the mutations are causative or whether the diabetes is a coincidental condition. In case series of patients with mutations in *RRM2B* and *OPA1*, the prevalence of diabetes was 3.2% and 3.5%, respectively (50, 51), comparable with the prevalence of diabetes in the background population. A higher proportion (11%) of patients with mutations in *POLG* had diabetes (52), but unlike MIDD, these patients invariably have severe complex, multisystem disease of which diabetes is a coexisting rather than presenting feature.

#### Diabetes complications and MIDD

High prevalence rates of peripheral neuropathy (58%), diabetic retinopathy (62%), and nephropathy (56%) were reported in Japanese patients with MIDD resulting from the m.3243A > G variant (33). A subsequent study of patients with the m.3243A > G variant compared the prevalence rates between those with and without diabetes (25). Peripheral neuropathy was much more common in those with diabetes (58% vs 8%). The prevalence of renal impairment was also higher in the patients with diabetes (13% vs 0%), and these patients presented with diabetes at a significantly lower mean age compared with those with normal renal function (26 vs 38 years). Diabetic eye disease was restricted to the patients with diabetes. No correlation was found between the variant load and the development of diabetic complications. A key question is whether the presence of the m.3243A > G variant modulates the risk of developing diabetes-related complications. This was addressed in a study of 74 patients with MIDD and the m.3243A > G variant and 134 diabetic control patients without the variant matched for factors known to influence complication risk (53). After correcting for HbA1c levels and hypertension, the prevalence of diabetic eye disease was decreased and that of renal dysfunction increased in patients with the m.3243A > G variant compared with the diabetic controls. These findings led the authors to postulate that the m.3243A > G variant modulates the risk of diabetes complications independently of known risk factors. The increased prevalence of renal dysfunction in patients with diabetes with the m.3243A > G variant was in line with an earlier report in Japanese patients (54). This increased prevalence might reflect the combination of diabetic nephropathy and focal segmental glomerulosclerosis (FSGS) resulting from the m.3243A > G variant. This is supported by the observation that all 3 patients with MIDD with moderate to severe renal dysfunction had evidence of FSGS on renal biopsy (24). Whether the m.3243A > G variant directly modulates the development of diabetic renal disease remains unanswered.

#### Pathophysiology of mitochondrial diabetes

Mitochondrial OXPHOS represents a key step in glucose stimulus-insulin secretion coupling. Mitochondrial DNA depletion leading to decreased mitochondrial respiratory subunit expression and decreased oxidative function has been shown to impair glucose stimulated insulin secretion in murine cell lines (55). However, the development of diabetes, as a consequence of mitochondrial dysfunction, does not appear to be limited to impaired stimulus-secretion coupling. Nuclear encoded mitochondrial transcription factor A (TFAM) is required for mtDNA biogenesis and maintenance (56). A pancreatic beta-cell TFAM knockout mouse model was created that resulted in tissue-specific mitochondrial dysfunction (56). Diabetes developed from the age of 5 weeks. Pancreatic islets from young mice revealed decreased glucose-stimulated insulin secretion (GSIS), cytochrome c oxidase depletion consistent with decreased mitochondrial subunit expression, and normal beta-cell mass. Diabetes persisted with increasing age, but islets from older (39-week-old) mice showed extensive beta-cell loss. However, the remaining beta cells showed no evidence of a severe defect of OXPHOS as determined using the histochemical reaction for cytochrome c oxidase, suggesting preferential loss of those cells with greater mitochondrial dysfunction (56). *TFB1M* and *TFB2M* are involved in mtDNA translation and transcription, respectively (57, 58). Isolated pancreatic islets from knockdown mice models of these nuclear-encoded genes both exhibited decreased GSIS, with evidence of decreased beta-cell mass in the *TFB2M* knockout mice (58). Taken together, these observations from animal models suggest that diabetes results from a combination of mechanisms that include decreased beta-cell mass in addition to impaired glucose stimulus insulin secretion coupling.

In the original description of the m.3243A > G variant and maternally inherited diabetes (21), it was reported that isolated skeletal muscle mitochondria from carriers of the mutation exhibited decreased mitochondrial subunit function and impaired OXPHOS. However, there are limited studies of human pancreatic tissue patients with diabetes because of the m.3243A > G variant. A decrease in beta-cell mass is a consistent finding (59, 60), with no evidence of increased apoptosis (59). Two studies explored the level of m.3243A > G heteroplasmy in pancreatic islet tissue. Lynn and colleagues found levels 10% to 45% in pancreatic islets from a patient with non-insulin-dependent diabetes mellitus and mitochondrial encephalomyopathy, lactic acidosis, and stroke-like episodes (MELAS) syndrome, with the expected high levels in skeletal muscle and brain (61). In this patient, there was no evidence of a severe OXPHOS deficiency in beta cells. Conversely, examination of pancreatic islet tissue from a patient who had insulin-dependent diabetes identified the presence of cytochrome c oxidase deficiency in beta cells and higher levels of variant averaging 63% heteroplasmy (60). The variability in the degree of heteroplasmy between studies may reflect different stages of the disease process in line with the observations from the TFAM mouse model.

Decreased GSIS is a consistent and primary pathophysiological feature of patients with m.3243A > G MIDD (23, 31). Conversely, the insulin secretory response to arginine was found to be normal (62, 63), consistent with direct triggering of beta-cell depolarization that is downstream of mitochondrial oxidative phosphorylation.

Insulin resistance (decreased insulin sensitivity) is a complex trait with genetic and nongenetic determinants, in particular adiposity, physical activity, and glucolipotoxicity (64). Decreased whole body sensitivity has been documented in carriers of the m.3243A > G variant (65, 66), with evidence of decreased insulin stimulated glucose uptake into skeletal muscle (65) and adipose tissue (67). However, the majority of the m.3243A > G carriers had abnormal glucose tolerance compared with the normal glucose-tolerant healthy controls, and so a confounding effect of hyperglycemia on insulin sensitivity cannot be excluded.

More recently, the advancement of induced human pluripotent cell technology derived directly from patient tissues (most commonly fibroblast) offers a promising avenue of exploring disease mechanisms of pathogenic mtDNA variants and an alternative to the animal model of investigating potential therapies (68). Several research groups have successfully generated induced human pluripotent cells and derived neurons using fibroblasts of patients with m.3243A > G-related MELAS and MIDD (69-72). These cell lines recapitulate the OXPHOS dysfunction, especially complex I deficiency identified in human tissues (69), and they have been used to study cell- and tissue-specific manifestations of m.3243A > G as well as the impact of different mutant heteroplasmy on cellular functions (69, 72).

In summary, impaired pancreatic beta-cell function plays a pivotal role in the development of abnormal glucose tolerance in MIDD, with a potential modulating effect of decreased whole body insulin sensitivity that may well result from secondary factors such as hyperglycemia and decreased exercise capacity.

### Short stature

Short stature is commonly associated with mitochondrial diseases. Boal et al reported a cohort of 575 adult patients with mitochondrial disease of different genotypes in the United Kingdom who were significantly shorter than their peers in the general population with a mean SD for height of -0.49 (95% CI, -0.58 to -0.39) (73). About 1 in 10 of adults with mitochondrial disease had height of below 2 SD of the population mean. Among them, those who harbored the m.3243A > G variant had significantly lower final height after adjusting for multiple comparisons. Short stature has also been reported in other forms of mitochondrial disease (74-77).

There are several explanations on how mitochondrial dysfunction affects growth and final adult height. First, mitochondrial dysfunction can adversely affect fetal and placental growth (78). At birth, infants with mitochondrial disorders have been shown to be significantly lighter than their healthy comparison groups (79, 80). In particular, babies born to mothers who harbored the m.3243A > G variants have lower birth weight (mean 2779 g; 95% CI, 2529-3029) compared with other mitochondrial genotypes (mean 3313 g; 95% CI, 3170-3456) and controls (mean 3429 g; 95% CI, 3314-3545) (81). Second, growth during early childhood is influenced by the interaction of many factors that include nutrition and coexistence of chronic disease. In patients with mitochondrial disease, reduced oral intake can be due to dysphagia (82, 83), gastroparesis, and intestinal pseudo-obstruction (84). Furthermore, coexisting chronic conditions such as diabetes mellitus and renal impairment can also restrict growth potential (48, 49). Short stature is also influenced by reduced muscle mass and lack of mobility in patients with early-onset mitochondrial disease. Neuromuscular manifestations of mitochondrial disease affect skeletal integrity and longitudinal bone growth. GH deficiency has been reported as the main cause of short stature in case reports, especially children who harbored the single, large-scale mtDNA deletion and m.3243A > G variant (85-88). However, these reports need to be balanced against those in which GH deficiency was not detected (89, 90). From a clinical perspective, GH replacement should be considered in those with clear evidence of biochemical deficiency because there have been favorable treatment outcomes in some (85, 91, 92) but not all patients (93).

### Hypoparathyroidism

Hypoparathyroidism has been reported in relation to Kearns-Sayre syndrome resulting from sporadic, single mtDNA deletions (94). In an older study, Harvey and Barnett studied 14 patients with Kearns-Sayre syndrome and clinical evidence of hypoparathyroidism and compared them with 212 patients without hypoparathyroidism (76, 95). The authors showed that although short stature and gonadal dysfunction with equal distribution between sexes were common features in this cohort, thyroid dysfunction, hyperaldosteronism, and hypomagnesaemia were rather uncommon (76). However, that might have been possibly because these abnormalities had not been properly looked for in some of the cases and concluded that higher prevalence of metabolic or endocrine abnormalities in patients with hypoparathyroidism might be, in fact, attributed to increased recognition rather than true clinical connection. In autopsy specimens, parathyroid glands are atrophic or even absent (96). Yet, in more recent reports, hypomagnesemia, which is commonly associated with hypoparathyroidism from suppression and/or impaired function of parathyroid hormone, has been described in few case reports (97, 98).

### Thyroid disease

A recent study analyzed data from the North American Mitochondrial Disease Consortium Patient Registry (99) and reported a prevalence of hypothyroidism of 6.3% based on a sample of 352 individuals with a confirmed molecular diagnosis of mitochondrial diseases. Although the definition of hypothyroidism was broad (combining clinical and biochemical based data), the authors commented that the prevalence rate of hypothyroidism was close to that for the general US background population.

### Hypoadrenalism

Primary hypoadrenalism has been reported in association with mitochondrial disease. A recent review of the literature identified 14 patients with mitochondrial disease and adrenal insufficiency; of these patients, 10 harbored mtDNA deletions, 2 patients harbored recessive *POLG* variants, 1 harbored recessive *GFER* variants, and 1 carried the m.8344A > G pathogenic variant (100). When reported, adrenal antibody titers have been negative (101). Mutations in other nuclear-encoded genes can impair mitochondrial biogenesis and have been associated with adrenal insufficiency (100). Chronic and episodic electrolyte disturbance can be a feature of mitochondrial disease (102), with adrenal insufficiency being a potential cause along with other factors such as renal tubular dysfunction.

### Fertility and hypogonadism

In view of the endocrine problems seen in patients with mitochondrial disease, there are potential issues with fertility (either male or female). The importance of this is difficult to evaluate for some pathogenic variants because of the relatively small numbers of patients, but the investigation of larger cohorts of patients has been invaluable in assessing the effect of the more common pathogenic variants. In a study looking at fertility among women aged 15 years or older from a large UK cohort of mitochondrial patients, the fertility rate was compared with the general population (with data obtained from the UK Office for National Statistics). Perhaps surprisingly, pathogenic mtDNA variants had no significant effect on the female fertility rate (carriers of mtDNA variants had a rate of 63.2 live births per 1000 person-years, compared with 67.2 live births per 1000 women in the general population; *P* = 0.36) (103). In addition, there was no significant difference in fertility rates between the most severely affected carriers and a comparable group of the general population. Fertility has also been explored in male patients with mitochondrial disease, and it has been proposed that mtDNA variants or nuclear mitochondrial gene variants affect sperm motility. Looking at male fertility in a clinical setting is more difficult than female fertility because of issues of paternity. However, a recent study looked at the reproductive success of men with mitochondrial disease and that of men in the general population. The reproductive success in mitochondrial patients was only 65% of the population, although many factors could contribute including severity of disease affecting attractiveness of male patients as partners, as well as direct sperm motility issues.

Although the data on populations of mitochondrial patients is helpful in terms of overall fertility, it is well recognized that pathogenic variants in certain genes can affect the functional activity of the gonads in both males and females (49). Hypogonadism is a well-recognized feature of mitochondrial disease, and both hypergonadotrophic and hypogonadotrophic hypogonadism has been reported. About one-fifth of patients with single large-scale deletions of mitochondrial DNA showed evidence of clinical and/or biochemical hypogonadotrophic hypogonadism, with equal prevalence between sexes (76). In a patient with Kearns-Sayre syndrome and hypopituitarism, treatment with human chorionic gonadotrophin resulted in increased testosterone levels and improvement in the secondary sexual characteristics (104), suggesting that early recognition and management may restore biochemical derangement and result in long-term clinical benefits (105). Hypergonadotrophic hypogonadism has been reported in the context of mitochondrial neurogastrointestinal encephalopathy (MNGIE) (106, 107) and/or infantile onset spinocerebellar ataxia caused by recessive variants in the *TWNK* gene, which encodes for mtDNA helicase Twinkle (108). In a cohort of patients with leukoencephalopathies associated with variants in *AARS2*, all female patients developed primary or secondary amenorrhea from ovarian failure before the age of 30 years (109). One of the most common nuclear gene-related mitochondrial disease, dominant variants in the *POLG* gene (110), have been linked with primary ovarian insufficiency (111) and primary testicular failure (112).

The findings of gonadic dysgenesis in males or females along with sensorineural hearing loss should raise suspicions of Perrault syndrome, a rare autosomal recessive mitochondrial disorder. In majority of patients with Perrault syndrome, their molecular genetic diagnoses remain undetermined (113, 114). To date, pathogenic variants in 6 different genes have been identified in Perrault syndrome, 5 of which encode mitochondrial related proteins—*TWNK, CLPP, LARS2, HARS2,* and *ERAL1. CLPP* encodes a mitochondrial ATP-dependent chambered protease (115), *LARS2* encodes mitochondrial leucyl-tRNA synthetase (116), *HARS2* encodes mitochondrial histidyl-tRNA synthetase (117), and *ERAL1* encodes a mitochondrial rRNA chaperone (118). These 5 genes that disrupt the mitochondrial homeostasis by altering its protein synthesis and degradation are widely believed to have led to the Perrault syndrome phenotype (114). Although the sixth gene associated with Perrault syndrome, the *HSD17B4* gene encodes a 17-beta-estradiol dehydrogenase in the peroxisomal fatty acid beta-oxidation (119). The prelingual onset of sensorineural hearing loss can be profound in early childhood and is progressive without evidence of vestibular impairment (113, 120). Gonadic dysgenesis can vary from primary amenorrhea to primary ovarian failure in females, whereas azoospermia has been reported in males (113, 114). Additional neurological features have been associated with Perrault syndrome, namely cerebellar ataxia, sensory peripheral neuropathies, intellectual disability, and leukodystrophy (114, 121, 122). Although there are fewer than 100 patients reported with a range of clinical features, under ascertainment of true prevalence is common because males without an affected sister are likely to be categorized as nonsyndromic hearing loss. Furthermore, hypogonadism may be underdiagnosed in patients with mitochondrial disease when other systemic manifestations predominate (94). Therefore, regular evaluation of the pituitary-gonadal axis hormones as well as clinical examination of these patients as part of their systemic clinical assessment will help to avoid long-term comorbidities.

### Pregnancy

During pregnancy, there are major endocrine and physiological changes that may either precipitate a worsening of a preexisting endocrine problem or the development of a new endocrine problem such as gestational diabetes. A systematic review published in 2011 (123) identified relatively few case reports of problems during pregnancy in patients with mitochondrial disease but they noted that there were no cohort studies. More recently, there have been 2 cohort studies that have looked more specifically at the effect of m.3423A > G variant on pregnancy outcome. One of these found that pregnancies of women with the m.3243A > G variant had significantly more gestational diabetes (16% of women). More than one-third of the pregnancies in women with m.3243A > G reported high blood pressure compared with those in the comparison group to international comparative studies of 3.6% to 9.1%. Only one-half of the pregnancies in the m.3243A > G group had normal vaginal delivery, with emergency cesarean section accounting for approximately 25% of deliveries. Babies were born earlier to mothers harboring m.3243A > G, with 53.3% of them preterm (<37 weeks). In the other cohort study of patients with m.3243A > G, 25.3% had a premature delivery and 5.5% had a gestation of ≤ 32 weeks, 12% suffered from preeclampsia, and 11% developed gestational diabetes. Based on this evidence, pregnant women with the m.3243A > G variant are at increased risk of antenatal complications, and it is therefore imperative that these women are under close combined obstetric and medical supervision during pregnancy. Screening for gestational diabetes is detailed in our online resource (124) and is in line with UK National Institute for Health and Care Excellence guidance (125).

For most other genetic defects causing mitochondrial disease, the size of the cohorts has limited our ability to assess the effect of pregnancy. However, there is now good evidence that, for women with pathogenic variants of *POLG,* there is a high incidence of disease onset or deterioration during pregnancy that can often lead to life-threatening consequences (126).

## Clinical Features—Nonendocrine

**Figure 2.**
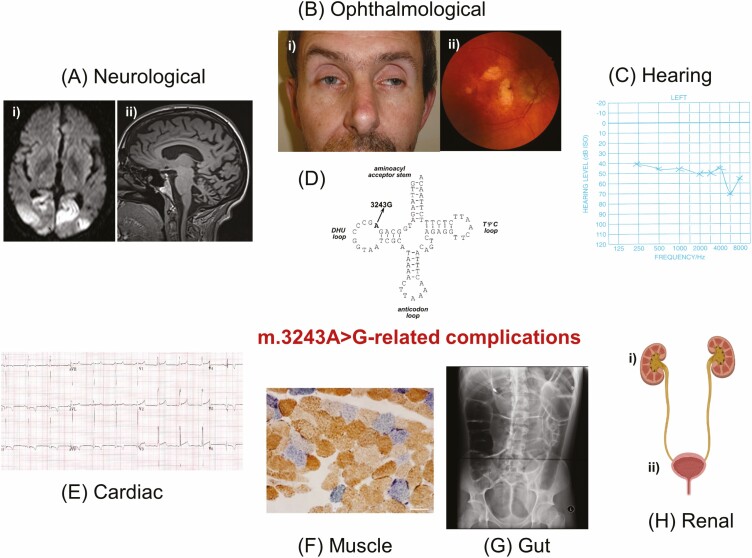
Multisystem manifestations of the m.3243-A > G-related mitochondrial disease. (A) Neurological. (i) Axial cranial MRI shows stroke-like lesions involving both occipital lobes with restricted diffusion. (ii) MRI sagittal view shows cerebellar and occipital lobe atrophy. (B) Ophthalmological. (i) Bilateral eyelid ptosis, reduced horizontal eye movement (ophthalmoplegia) and overactive frontalis muscles. (ii) Retinal picture shows macular dystrophy. (C) Audiological. Audiogram reveals a moderate to severe hearing loss that is more severe in the high-frequency range. (D) The position within the variable DHU loop of mt-tRNA^Leu(UUR)^ where the A > G substitution occurs. (E) Cardiac. A 12-lead ECG showing short PR interval (Wolff-Parkinson-White syndrome) and increased LV voltage compatible with LVH with repolarization abnormality. (F) Muscle. A skeletal muscle biopsy demonstrating COX-deficient muscle fibers (blue fibers). (G) Gut. Abdominal radiography showing severe dilated large bowel without evidence of mechanical blockade, consistent with intestinal pseudo-obstruction. (H) Renal. (i) Focal segmental glomerulosclerosis, tubulopathy, and chronic kidney disease are recognized associations with the m.3243A > G variant, independent of the diabetic status. (ii) Lower urinary tract dysfunction such as detrusor overactivity, bladder outlet obstruction, and stress incontinence has been reported. COX, cytochrome c oxidase; ECG, electrocardiogram; LV, left ventricular; LVH, left ventricular hypertrophy; MRI, magnetic resonance imaging.

The clinical phenotype of mitochondrial disease is highly heterogeneous because patients can present any age group and organ involvement (17). Many mitochondrial syndromes have been reported in the literature, and such syndromic descriptions have undoubtedly aided the pattern recognition and testing for mitochondrial disease (127). However, it is increasingly recognized that most patients do not present with a classic syndrome, and the overall clinical phenotype of individuals often evolve with time. Some genetic defects are prevalent in the general population, such as m.3243A > G variant and single, large-scale mtDNA deletion (16), and endocrinologists are likely to encounter these patient groups in clinical practice, presenting with endocrine abnormalities and other multisystem features (**Table 1** and **Fig. 2**). In this section, a general overview of different systemic manifestations is outlined.

**Table 1.** Summary of multisystem disease and clinical management in the m.3243A > G variant and single, large-scale mtDNA deletion

	m.3243A > G	Single, large-scale mtDNA deletion	Surveillance and management
Inheritance	Maternally inherited	Mostly sporadic	• Genetic counselling • Reproductive options
Endocrine involvement			
Diabetes mellitus	Common (~50%)	10%-15%	• Periodic assessment for diabetic complications and CVD risk factor management • Pharmacological treatment algorithm (**Figure 4**)
Short stature	Common	Especially in those with childhood-onset disease (KSS)	• Likely multifactorial • Optimize nutrition • Consider investigating GH deficiency in patients with childhood-onset disease
Hypoadrenalism	Very rare	Especially in those with childhood-onset disease (KSS)	• Exclude autoimmune etiology • Steroid replacement
Hypoparathyroidism	Very rare	Especially in those with childhood-onset disease (KSS)	• Calcium supplementation, vitamin D replacement, thiazide diuretics, phosphate binders
Audiology			
Sensorineural hearing loss	Very common	Very common	• Audiology testing if symptomatic • Hearing aids • Consider cochlear implant for some cases (128)
Neurological			
Stroke-like episodes	~20% (The clinical and radiological changes are distinctive from thromboembolic stroke)	N/A	• Aggressive seizure treatment • MRI of the head, EEG • Details of investigation and management of stroke-like episodes are available elsewhere (129)
Leukodystrophy	N/A	Especially in those with childhood-onset disease (KSS) (130)	• MRI of the head • Consider testing for CSF 5MTHF • Consider a trial of folinic acid (131)
Myopathy/exercise intolerance	Common	Common	• Pacing for physical activities • Exercise is safe and beneficial (132) • Monitor for lactic acidemia
Ophthalmological			
CPEO	Common	Very common	• Referral to oculoplastic surgeon for eyelid corrective surgery • Consider prism for diplopia
Retinal changes	Maculopathy (133); pigmentary changes	Pigmentary changes	• Monitor for change in visual acuity/night vision • May coexist with diabetic retinopathy
Cardiac	Common (~25%) Structural: LVH, HCM, heart failure Electrophysiology: pre-excitation, WPW	Especially in those with childhood-onset disease (KSS) Structural: LVH, HCM, heart failure Electrophysiology: bi-/trifascicular heart block, complete heart block	• Annual ECG and echocardiogram • If cardiac screening is abnormal, refer to cardiologist for further assessment and treatment (eg, treatment for LVH/HCM, electrophysiology study/ablation, PPM) (134)
Gastrointestinal	Chronic constipation is very common; intestinal pseudo-obstruction can occur concomitantly with stroke-like episodes	Chronic constipation is very common	• Regular laxatives • Surgical resection is NOT indicated in most cases; details of investigation and management of intestinal pseudo-obstruction are available elsewhere (84, 135)
Renal	Focal segmental glomerulosclerosis; renal tubular acidosis (Fanconi syndrome); end-stage renal failure	Renal tubular acidosis; end-stage renal failure	• Annual screening for urea, electrolytes and proteinuria • The kidney disease could occur and progress independently of diabetes mellitus • Referral to nephrologist for the management of chronic kidney disease • Renal transplant may be required for some cases

Abbreviations: 5MTHF, 5-methyltetrahydrofolate; CPEO, chronic progressive external ophthalmoplegia; CSF, cerebrospinal fluid; CVD, cardiovascular disease; EEG, electroencephalogram; HCM, hypertrophic cardiomyopathy; LVH, left ventricular hypertrophy; KSS, Kearns-Sayre syndrome; MRI, magnetic resonance imaging; N/A, not applicable; PPM, permanent pacemaker; WPW, Wolff-Parkinson-White syndrome.

### Neurological

Approximately 80% of all patients with mitochondrial disease have evidence of neurological involvement (99). Some neurological involvements are common but not specific, such as neurodevelopmental delay in children, myopathy, and exercise intolerance in adults. In contrast, some neurological findings are strongly indicative of specific mitochondrial etiology. They are the canonical feature of syndromic diagnoses, such as sequential painless visual loss in Leber hereditary optic neuropathy, developmental regression and symmetrical basal ganglia signal abnormality in Leigh syndrome, and stroke-like episodes in MELAS syndrome. More in-depth review of neurological presentations of mitochondrial disease is available elsewhere (1, 136).

### Cardiac

The spectrum of cardiac involvement in mitochondrial disease includes structural heart abnormality, for instance, cardiomyopathy, Wolff-Parkinson-White syndrome caused by preexcitation accessory pathway, and variable degrees of heart block (134). Cardiac abnormalities such as asymptomatic left ventricular hypertrophy and Wolff-Parkinson-White are often insidious at the outset. They frequently form part of multisystem disease identified via the cardiac screening in common mtDNA variants such as m.3243A > G and m.8344A > G (137). Progressive conduction defects ranging from first-degree heart block to complete heart block appear to be more specifically associated with patients with single, large-scale mtDNA deletion manifesting with Kearns-Sayre syndrome. A recent retrospective study of 260 patients showed that patients with m.3243A > G and single, large-scale mtDNA deletion were associated with the highest risk of major adverse cardiac events, including heart failure, cardiac arrest, and sudden death (138). On the other hand, cardiac manifestations could be the most prominent presenting feature (ie, Senger syndrome caused by recessive *AGK* variants (139) and homoplasmic m.4300G > A variant (140)). Early recognition of cardiac abnormalities and therapeutic intervention is crucial because cardiac death remains the most common cause of death in adult mitochondrial disease from pathogenic mtDNA variants (141-144). However, it is important to highlight that the risk of cardiac manifestation is genotype specific. It is rare in many common nuclear gene defects linked to chronic progressive external ophthalmoplegia and mtDNA maintenance disorder in adults (145).

### Gastrointestinal

Symptoms of gastrointestinal tract (GIT) dysmotility such as early satiety, lack of appetite, bloated feeling, and chronic constipation are relatively common in adult patients with mitochondrial disease (83). Some patients develop severe gut dysmotility, manifesting as gastroparesis and intestinal-pseudo-obstruction (146), which are potentially life-threatening if not diagnosed and managed promptly. A UK cohort study identified that intestinal-pseudo-obstruction could develop concomitantly with stroke-like episodes in 50% of m.3243A > G cases (84). MNGIE syndrome resulting from thymidine phosphorylase deficiency is an ultra-rare mitochondrial syndrome also characterized by prominent manifestations of GIT dysfunction such as cachexia, malnutrition, severe gut dysmotility, and other neurological features including chronic progressive external ophthalmoplegia, demyelinating neuropathy, and asymptomatic leukodystrophic changes identified on cranial magnetic resonance imaging (147, 148).

In hepatocerebral syndrome secondary to mtDNA depletion (4), liver disease is severe and frequently manifests with hypoglycemia, deranged liver function test, coagulopathy, and lactic acidosis in early childhood, typically during infancy. The liver disease could progress rapidly to fulminant hepatic failure requiring liver transplantation. These children frequently have progressive neurological involvements, such as generalized hypotonia, neurodevelopmental delay, and muscle weakness, some with neuropathy and intractable epilepsy. The outcomes of hepatocerebral syndrome and liver transplantation have been variable, in part, are determined by the underlying genetic defect (149, 150). For example, some patients with *POLG*-related Alpers disease survived the liver transplantation but succumbed to super-refractory status epilepticus (151).

### Renal

Renal involvement forms a part of the clinical phenotype in some genetic defects causing mitochondrial disease (152). The examples of kidney disease are steroid-resistant nephrotic syndrome, FSGS, tubulopathy including proximal and distal renal tubular acidosis, and cystic changes with hypertension (153). In cases of m.3243A > G variant, chronic kidney disease can emerge independent of the diabetic status (154) or precede the development of diabetes (155). Some patients with renal involvement can progress into end-stage renal failure, and anecdotal observation would suggest that their outcome with renal replacement therapy, including transplantation, is encouraging (150, 153, 155).

### Other organ involvements

Although mitochondria are ubiquitous, the tissue specificity associated with certain genetic defects remains fascinating and puzzling in clinical practice. Ekbom syndrome, characterized by multiple symmetric lipomatosis, myoclonus, ataxia, and neuropathy, is strongly associated with m.8344A > G and myoclonic epilepsy and ragged red fibers syndrome but has rarely been reported in other pathogenic mtDNA and nuclear DNA variants (156), except in cases of recessive *MFN2* variants (157). Sideroblastic anemia is an uncommon presentation of mitochondrial disease and has only been consistently observed in a handful of genetic defects, such as single, large-scale mtDNA deletion, *PUS1* and *YARS2* (158, 159).

## Diagnosis

Endocrinologists play an important role in providing guidance on the investigation and management of endocrine complications related to the mitochondrial disease. On the other hand, several scenarios should prompt endocrinologists to consider whether there is primary mitochondrial etiology for “atypical” presentations of endocrine problems such as diabetes mellitus, short stature, adrenal insufficiency, and premature ovarian failure.

### Diagnostic approaches for mitochondrial disease

There have been many recent advances in genetics that have greatly simplified the diagnosis of mitochondrial disease and other genetic diseases causing endocrine problems. Establishing a genetic diagnosis is crucial in view of the potential complications of mitochondrial disease and providing accurate genetic counselling. The advent of next-generation sequencing (NGS) has made the biggest change, with patients with mitochondrial disease being identified using gene panels or more recently by whole exome (WES) or whole genome sequencing (WGS) (5).

Although there has been progress with mitochondrial biomarkers in terms of detecting neurological features of mitochondrial disease, particularly neuromuscular involvement, the use of biomarkers in terms of endocrine features is still of unknown value. The 2 most widely studied biomarkers are fibroblast growth factor-21 (160) and growth differentiation factor 15 (161), but both are best for detecting muscle involvement. Other metabolomics and proteomic biomarkers are being explored at present but are not yet in clinical practice (162, 163).

#### Mitochondrial DNA genomics

There is considerable variability in the availability of specific genetic tests in individual countries. Screening for specific prevalent pathogenic variants such as m.3243A > G by techniques such as pyrosequencing is still practiced in some laboratories, whereas in others testing proceeds straight to amplification of mtDNA for NGS. The high depth of coverage in such sequencing provides more sensitive detection of single nucleotide mtDNA variants and accurate quantitation of mutant load. Large-scale mtDNA rearrangements are also detected but if accurate quantitation is required, alternative approaches such as quantitative PCR should be used. Some centers also extract mtDNA sequences from WES data, although this is less reliable than the other methods described because of a relatively low depth of sequencing (164, 165).

There has to be great care in testing for pathogenic mtDNA variants in blood alone. This can be due to the marked tissue segregation of some pathogenic variants (for example, single, large-scale mtDNA deletions) and the decline of heteroplasmy of specific variants in blood over time from active selection (for example, the m.3243A > G variant (13)). In addition, the m.3243A > G variant is present at low levels in blood in approximately 1 in 250 in the population, so very low levels detected on NGS may not indicate m.3243A > G disease. For m.3243A > G, the use of additional, noninvasively obtained tissues such as urinary sediment for screening mtDNA variants is encouraged (166). If mtDNA screening is uninformative in blood- and urine-derived DNA, subsequent testing of muscle may be needed to fully exclude mtDNA disease (for example, single, large-scale mtDNA deletions and de novo single nucleotide variants (167)).

#### Nuclear DNA genomics

Pathogenic variants in more than 300 nuclear genes cause mitochondrial disease (5), although as commented previously, relatively few of these are associated with endocrine abnormalities. However, because more than 1100 mitochondrial proteins have been identified, it is highly likely that new genetic defects will be identified. In many laboratories, unbiased WES and WGS approaches have replaced the testing single genes. The diagnostic yield of WGS is usually greater than WES because of improved detection of mtDNA sequences and copy number variants, and the ability to study noncoding region. An important part of the WGS or WES pathway is the selection of the gene list to be analyzed and whether to use a clinical exome, for example. The practices vary in different countries and potentially between laboratories, especially those with a particular research interest. In some diagnostic laboratories, there is only a narrow phenotype-defined gene list, whereas others are using lists containing all genes causing inherited disease. As with other genetic diseases, understanding whether rare variants are pathogenic remains a challenge but the widespread adoption of the American College of Medical Genetics criteria for variant classification and the development of specific algorithms are helping (1).

### MIDD Diagnostic Algorithm

**Figure 3.**
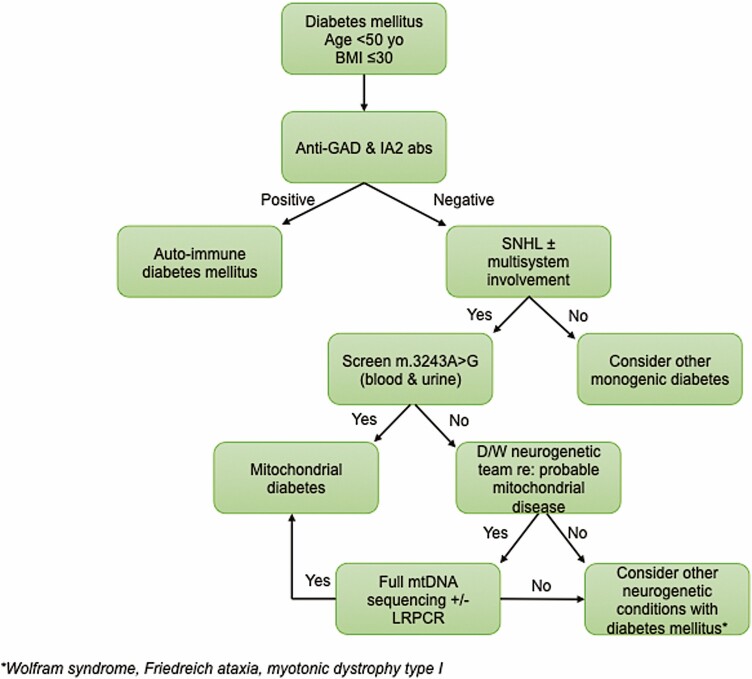
Proposed diagnostic algorithm for mitochondrial diabetes. LRPCR, long-range PCR; SNHL, sensorineural hearing loss.

Diabetes is invariably an early clinical manifestation of MIDD, and as such offers the first opportunity for detection and diagnosis of mitochondrial disease. We have created an algorithm (**Fig. 3**) that provides an approach and guidance to the investigation of diabetic patients who may have mitochondrial diabetes. As detailed previously, MIDD usually presents in middle age and should be considered when diabetes presents before 50 years of age. Patients are invariably nonobese, although in patients with established diabetes there may have been subsequent weight gain secondary to their diabetes therapy. In line with the recommendations of an expert forum for the diagnosis of monogenic diabetes (29), it is crucially important to identify and to exclude patients who have autoimmune diabetes as they have clear and specific therapeutic and management needs.

The combination of diabetes and sensorineural deafness is a strong signal for MIDD. However, a small proportion of patients will have no hearing impairment, although they may have other neurological features of mitochondrial disease, and/or a maternal history of diabetes and deafness. It is worth noting, however, that a maternal history of diabetes alone is not a reliable signal for MIDD (45). Screening for the m.3243A > G variant is often performed using an EDTA blood sample, but the levels of heteroplasmy can be below the detection threshold in this tissue (13), so other tissues such as uroepithelial cells, or postmitotic skeletal muscle, may require analysis (1). If the m.3243A > G variant is not detected, then we would recommend discussion with the local neurogenetics team as screening for other mitochondrial DNA variants including single, large-scale mtDNA deletion may be indicated. If this is deemed unnecessary or fails to identify a causative mitochondrial DNA variant, other forms of monogenic diabetes (29) or neurogenetic disorders (77) may need to be considered (1).

## Management

A significant proportion of patients with mitochondrial disease are at risk of developing multisystem complications, their health care needs are complex, and frequently require access to multiple health professionals. The most challenging patients are often managed by specialist centers for mitochondrial disease (eg, the NHS Highly Specialised Service in the United Kingdom (168), mitochondrial medicine centers in the United States (169), and the European Reference Network of Neuromuscular Diseases (170)) that facilitate the delivery of multidisciplinary care together with the local primary and hospital services. These centers also offer guidelines on care (135, 171) and advice to clinicians working in other hospitals.

### Management of endocrine problems

The management of hormone deficiency in relation to mitochondrial disease is broadly straightforward and involves hormone replacement in line with standard endocrine practice.

The situation is more complicated for blood glucose management in patients with mitochondrial diabetes.

Patients presenting with clinical features consistent with insulin deficiency (weight loss and ketosis) should be managed in the same way as patients with autoimmune type 1 diabetes. Importantly, they should have access to the same educational support and metabolic monitoring systems including the noninvasive continuous glucose monitoring system.

Those presenting with non-insulin-dependent diabetes should be managed in accordance with type 2 diabetes guidance, but there are a number of considerations that influence patient management and selection of oral hypoglycemic agents:

1. Increased risk of progression to insulin deficiency. For this reason, we advocate a baseline C-peptide to gauge insulin secretory reserve and education in home ketone testing.
2. Because patients with mitochondrial diabetes are at risk of lactic acidosis, especially during acute ill health, we recommend the avoidance of metformin.
3. Patients with mitochondrial diabetes respond well to sulphonylureas, with some being particularly sensitive to recurrent hypoglycemia. We advocate therefore introducing sulphonylureas at a low dose and ensure that the patient is compliant with blood glucose monitoring.
4. Most patients with mitochondrial diabetes are nonobese and often experience GIT dysfunction that can contribute to impaired nutrition (see the Gastrointestinal section). To try to limit these problems, we would advocate dipeptidyl peptidase-4 inhibitors rather than glucagon like peptide-1 receptor analogues, although both have been reported to effective in a small case series (172).
5. Sodium-glucose transport protein 2 inhibitors have been reported to be both cardio- and reno-protective in type 2 diabetes (173), and it is assumed that these benefits extend to patients with mitochondrial diabetes, although there have been no randomized controlled trials. Nonetheless, this class of agents have been reported to improve glycemic control in mitochondrial patients (172). These agents impart an increased risk of ketoacidosis, and so education around ketone testing and sodium-glucose transport protein 2 cessation is critically important in patients with mitochondrial diabetes.

**Figure 4.**
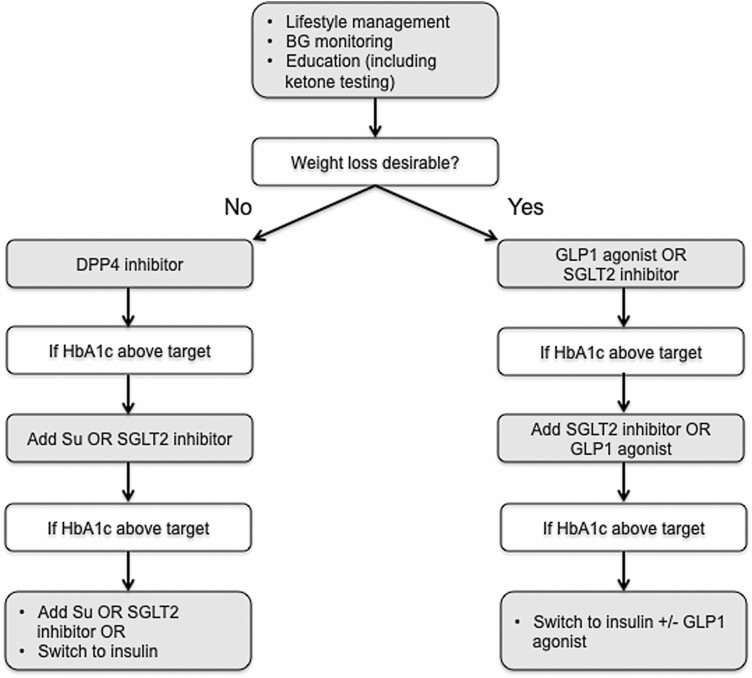
Proposed management algorithm of mitochondrial diabetes. BG, blood glucose; DPP4, dipeptidyl peptidase-4; GLP1, glucagon-like peptide 1; SGLT2, sodium-glucose transport protein 2; Su, sulphonylurea.

Based on these considerations, we propose a treatment algorithm for patients presenting with non-insulin-dependent mitochondrial diabetes (**Fig. 4**).

### General management of mitochondrial disease and surveillance for systemic involvement

With a few exceptions, the principle of clinical management of mitochondrial disease is supportive (**Table 1**). Several emergencies are associated with mitochondrial disease, including stroke-like episodes and refractory seizures, brainstem crisis in Leigh syndrome, and multiorgan dysfunction associated with severe metabolic acidosis (1). At-risk patients, their caregivers, and clinicians should be equipped with the knowledge of recognizing these potentially life-threatening scenarios so that prompt medical treatment, such as IV anticonvulsant for stroke-like episodes and appropriate monitoring and supportive measures, can be instigated (129).

Some clinical problems such as sensorineural hearing loss, cardiac involvement, and chronic gut dysmotility are progressive in nature. Regular review following the genetic diagnosis is necessary. There are effective interventions to ameliorate these symptoms and improve quality of life. Moreover, patients with positive cardiac screening results such as left ventricular hypertrophy and conduction defect should be referred to dedicated cardiac service for further management because of the potential severe outcome. Patients with significant central nervous system involvement or neuromuscular weakness are at risk of developing respiratory insufficiency and dysphagia; therefore, assessment of respiratory and swallowing function should be considered periodically. Given the complexity and heterogeneity of clinical manifestations in mitochondrial disease, the “1 size fits all” approach is impractical. Personalized surveillance strategies should be developed and refined based on the findings derived from natural histories of different genetic defects.

Despite lacking robust trial evidence of proven efficacy, multivitamins and cofactors supplementation, frequently referred to as “mito cocktails,” is widely promoted by some medical providers (174) and accepted by many patients and their caregivers. According to a US survey of patients with mitochondrial disease and their caregivers, around 75% of the responders took at least 4 different supplements even though similar proportion of responders reported no clinical benefits (175). The main reasons behind such empirical use of vitamin supplements and antioxidants include the perceived safety profile compared with prescribed medicines, overstated benefits in preclinical studies and case reports, and the absence of specific therapy for most forms of mitochondrial disease. However, the financial cost of “mito cocktails,” the physical burden of supplement intakes in addition to other prescribed medications among patients with swallowing difficulties or those who rely on gastrostomy tube, and potential harms identified in mouse models (176), are frequently underestimated. On the other hand, there are specific (and rare) circumstances that long-term supplementation of vitamin and supplement are clinically indicated (177, 178), such as in those with primary CoQ10 deficiency, riboflavin transporter deficiency, biotinidase deficiency, and cerebral folate deficiency.

### Clinical trials and experimental treatments

**Figure 5.**
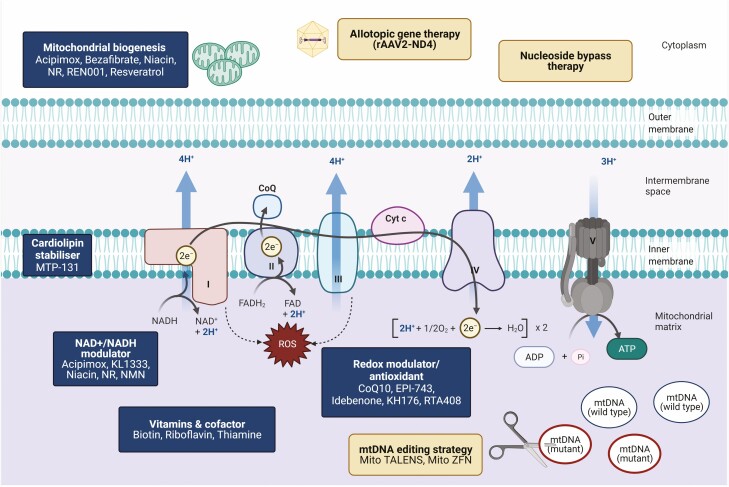
Emerging treatment strategies for mitochondrial diseases. Repurposed and novel small molecules targeting different mitochondrial pathways (mitochondrial biogenesis, cardiolipin stabilizer, NAD^+^/NADH modulator, redox modulator/antioxidant) are classified as generic treatment approaches that have the potential to translate across different types of mitochondrial disease. On the other hand, specific treatment approaches such as allotopic gene therapy for LHON, nucleoside bypass therapy for mtDNA depletion syndrome, and methods of eliminating pathogenic mtDNA variants are examples of precision medicine in the mitochondrial field. Created with BioRender.com.

There is considerable progress in finding better therapies for different forms of mitochondrial disease over the past decade (**Fig. 5**) (179-181). Idebenone, an analogue of CoQ10, has become the first licensed drug approved by European Medicine Agency specifically for treating visual impairment in patients with Leber hereditary optic neuropathy in Europe (182). More recently, a phase 3 randomized, controlled clinical trial involving a form of gene therapy, allotopic expression of ND4 gene, demonstrated a clinically meaningful improvement of visual function in patients who harbors the m.11778G > A variant, which accounts for > 70% of Leber hereditary optic neuropathy cases (183).

Small molecules that promote mitochondrial biogenesis or modulate the NAD^+^/NADH ratio are emerging as an important and generic treatment strategy for mitochondrial myopathy, irrespective of the underlying genetic defects (179). Several novel compounds or repurposed drugs have shown some promising results in phase 2 studies (184). However, phase 2 studies in mitochondrial disease have typically been open labelled with a small patient number. It is crucial that the therapeutic efficacy is robustly confirmed in the phase 3 trials (176).

Enzymatic defects of the nucleotide metabolism, such as deficiencies of thymidine phosphorylase and thymidine kinase 2, can lead to mitochondrial DNA depletion. Thymidine phosphorylase deficiency causes systematic accumulation of nucleosides, leading to MNGIE syndrome. There are 2 main treatment strategies (185): (1) direct removal of accumulated nucleosides through either hemodialysis or peritoneal dialysis; and (2) enzymatic replacement including platelet transfusion, allogenic hematopoietic stem cell transplantation, erythrocyte-encapsulated thymidine phosphorylase, and orthotopic liver transplant. Thymidine kinase 2 deficiency causes severe mitochondrial myopathy (186), and open-labelled studies of nucleoside therapy appeared to stabilize the disease and lead to functional improvement (187).

The onset and severity of some mitochondrial DNA diseases are determined by the mutant heteroplasmy. Two mitochondrially targeted programmable nucleases, namely the mitochondrial transcription activator-like effector nucleases or mitochondrial zinc finger nucleases, have been developed to eliminate the heteroplasmy of pathogenic mtDNA variant. Both methods have demonstrated the efficacy of reducing mutant heteroplasmy in cell lines and a mitochondrial tRNA mouse model (188, 189). However, there are inherent challenges in translating laboratory techniques into human studies, especially in patients who manifest with multisystem diseases, as the efficiency of delivering nucleases to different organs is likely to be variable.

## Reproductive Options

One area of mitochondrial disease that has attracted considerable attention recently is development of reproductive options for families with mitochondrial disease. It is particularly important because of the lack of curative treatment for patients and the advancements in genetics establishing a genetic diagnosis in most patients.

Broadly genetic advice is divided into 2 main groups—those patients with a nuclear gene pathogenic variant and those with involvement of the mitochondrial genome. For families with nuclear mitochondrial disease, advice will depend on the nature of the inheritance (for example, autosomal dominant or recessive) and in certain countries options such as prenatal testing and preimplantation genetic testing (PGD) are options. For families with pathogenic variants involving the mitochondrial genome, the advice is very different, reflecting the different inheritance of the mitochondrial genome.

**Figure 6.**
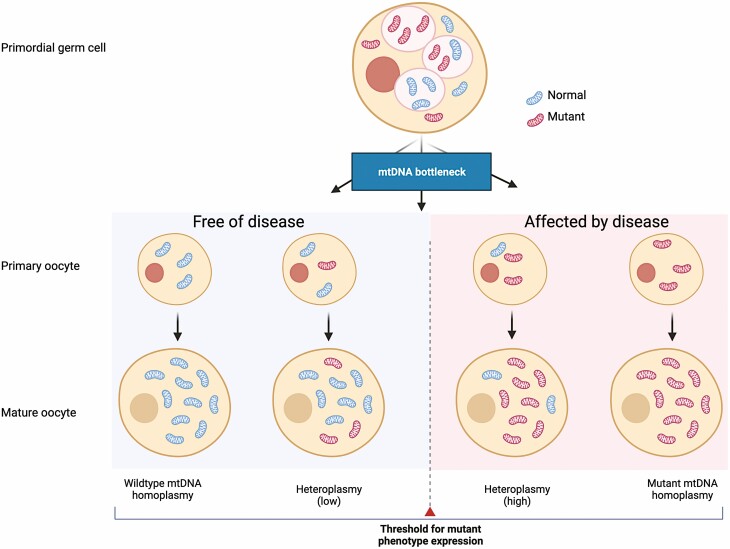
Mitochondrial DNA transmission. Homoplasmic variants are transmitted from mother to offspring. The mitochondrial bottleneck explains how there can be extreme divergence in the heteroplasmy between mother and offspring. There is a genetic bottleneck during development that results in different heteroplasmy in each individual oocyte. This is a major challenge when providing genetic counselling for mothers with heteroplasmic mtDNA variants because the level of heteroplasmy will determine the clinical outcome in the offspring. This figure is derived from a previous published work (1) and created with BioRender.com.

Mitochondrial DNA is maternally inherited and goes through a genetic bottleneck in development raising several issues as regards genetic counselling. In the presence of heteroplasmy, this transmission is complicated because the bottleneck leads to the potential for extreme variation in the level of heteroplasmy between the mother with the pathogenic mtDNA variant and her offspring (**Fig. 6**). This is very important when we consider the reproductive options for women with pathogenic mtDNA variants. All female carriers of pathogenic mtDNA are at risk of transmitting the mtDNA variant and thus mtDNA disease to their offspring. The risk very much depends on the mtDNA variant. For example, the majority of single, large-scale mtDNA deletions are sporadic so mothers with 1 affected child may well have other clinically unaffected children. However, there is still a small risk of transmission if a patient him- or herself has an mtDNA deletion (possibly as high as 1 in 20 (190)). For patients with the m.3243A > G variant, and many other pathogenic variants (191), the risks are very different with virtually all mothers transmitting the pathogenic variant to their offspring. For mothers with homoplasmic mtDNA defects, all offspring will inherit the pathogenic variant. (192) If a patient is male, then there have been no reports of paternal transmission of mtDNA disease; therefore, they can be reassured there is no risk of transmitting the disease.

### Reproductive options for women with pathogenic mtDNA variants

#### Prenatal diagnosis

Prenatal diagnosis involves testing the level of heteroplasmy in tissue obtained by chorionic villus sampling or amniocentesis. The level of heteroplasmy detected in the tissue is reported to reflect that seen in children (193). One of the major challenges is deciding what the risk is for a specific level of heteroplasmy for an individual variant. Where available, the results of mtDNA heteroplasmy analyses from other family members are helpful in interpreting the prenatal mtDNA test result especially if the variant is relatively rare. Prenatal diagnosis may be an option for women with low risk of transmission, for example, patients with single, large-scale mtDNA deletions (190), who want to ensure that there is no transmission. However, with any procedure there is a risk in the procedure itself (reported at around 1% loss of pregnancy), which needs to be considered (194).

#### Preimplantation genetic diagnosis

PGD involves the testing of embryos and then implanting an embryo with low level of heteroplasmy. PGD is certainly a good option for those women who have mtDNA variants that widely segregate in oocytes (195). In these patients, often some oocytes will have variant levels well below the level at which symptoms occur. For other heteroplasmic mtDNA variants, the challenges of PGD are much greater. This has recently been looked at in detail for the m.3243A > G variant to determine if the level in offspring can be predicted (196). The higher the mutation load in the mother, the less likely the offspring are to have levels at which disease is unlikely. It is also important to be aware that successful birth is heavily dependent on the quality of the embryo and the embryo with the lowest level of heteroplasmy might not be the best quality embryo.

#### Mitochondrial donation

**Figure 7.**
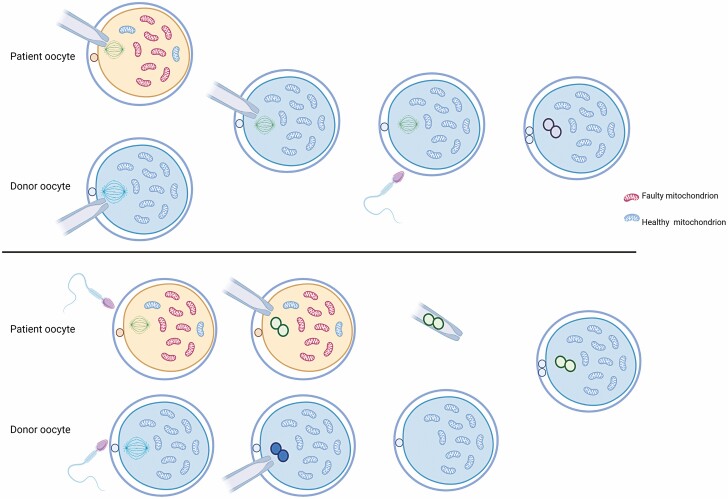
Mitochondrial replacement therapies. (A) Metaphase II transfer. (B) Pronuclei transfer. (A) Metaphase II transfer involves removing the metaphase II spindle from the donor oocyte and then transferring the metaphase II spindle from the patient carrying the pathogenic mtDNA variant. The oocyte is subsequently fertilized by the partner’s sperm. (B) Pronuclear transfer involves transferring the pronuclei, formed immediately after fertilization, from the mother’s oocyte into the enucleated oocyte of the donor woman. Both metaphase II spindle transfer and pronuclear transfer result in the nuclear DNA from both parents with the mitochondria (and mtDNA) from the donor woman. This figure is modified from a previous published work (197) and created with BioRender.com.

Recently, another potential option is available for women with pathogenic variants, mitochondrial donation (also called mitochondrial replacement therapy). The rationale for developing these in vitro fertilization (IVF) techniques was due to no options being available for women with homoplasmic or high heteroplasmic mtDNA variants to have their own biologically related child, with no risk of serious disease in the offspring. Several groups pioneered the experimental techniques in embryos using metaphase spindle transfer (MST), pronuclear transfer (PNT), and polar body transfer. Most experience in human embryos is with MST and PNT (**Fig. 7**). MST involves the transfer of the metaphase II spindle from the oocyte with the pathogenic variant into a donor oocyte with normal mitochondria (198). Following transfer, the oocyte is then fertilized. PNT is performed immediately after fertilization with the transfer of the male and female pronuclei from the single-cell zygote with the pathogenic mtDNA variant to the donor zygote with normal mitochondria (199). Both techniques have been evaluated using human oocytes and shown that following transfer development to clinical grade embryos is feasible.

### Providing a care pathway for families with pathogenic mtDNA variants

There are many challenges when considering the reproductive options for women with mtDNA variants. It is important to discuss reproductive options early with women with pathogenic mtDNA variants because fertility declines with age and the options become more limited with time. Advice should be individualized for the specific mtDNA variant (196) and it is very important to consider the mother’s health because of increased risks during pregnancy for some women (200).

Counselling women for women with pathogenic mtDNA variants should include all options including voluntary childlessness, adoption, and oocyte donation. Oocyte donation will prevent transmission of the mtDNA variant but is limited by availability of oocytes. For those women who wish to have their own biologically related child, the possible options, depending on the variant, are prenatal testing, PGD, and mitochondrial donation (mitochondrial replacement therapy). The availability of these different options is very dependent on what is allowed in individual countries. In view of these challenges, it is important that there is careful consideration of the care pathway available. As recently highlighted in the report on Hereditable Genome Editing from the USA Academies of Science and Medicine and the Royal Society, IVF regulation is variable in different countries (201). Some countries have a highly regulated system, whereas in others, there is little or no regulation, meaning that impartial advice can be difficult to obtain. In the UK, IVF is regulated by the Human Fertilisation and Embryology Authority and, following the passing of the Mitochondrial Donation regulations through the UK Parliament, has approved mitochondrial donation for clinical under strict regulations (202).

## Conclusion

A potential role of pathogenic mitochondrial gene variants in endocrine disease was first recognized in 1992, and there have been numerous publications documenting this in case reports. More recently, large national cohort studies have given a much better insight into the true role of mitochondrial dysfunction in endocrine disease. The major endocrine manifestation of mitochondrial disease is diabetes mellitus, and this is often accompanied by sensorineural deafness in patients with the m.3243A > G pathogenic variant. Other endocrine abnormalities are less common but include hypogonadism and infertility, adrenal insufficiency, and hypoparathyroidism.

Patients may present to the endocrinologist, and making a genetic diagnosis is important because this has significant implications for disease surveillance. The majority of patients with mitochondrial disease will have involvement of other organ systems and these can have life-threatening, but often treatable, manifestations. Establishing a genetic diagnosis also has consequences in terms of transmission of the pathogenic variant to offspring. Recent developments in reproductive technologies means that, for families with mitochondrial disease, reproductive options are available.

## Abbreviations

BMI: body mass index
FSGS: focal segmental glomerulosclerosis
GAD: glutamic acid decarboxylase
GIT: gastrointestinal tract
GSIS: glucose-stimulated insulin secretion
ICA: islet cell antibody
IVF: in vitro fertilization
MELAS: mitochondrial encephalomyopathy
lactic acidosis: and stroke-like episodes
MIDD: maternally inherited diabetes and deafness
MNGIE: mitochondrial neurogastrointestinal encephalopathy
MST: metaphase spindle transfer
NGS: next-generation sequencing
OXPHOS: oxidative phosphorylation
PGD: preimplantation genetic testing
PNT: pronuclear transfer
TFAM: mitochondrial transcription factor A
WES: whole exome sequencing
WGS: whole genome sequencing

## Additional Information

**Disclosures:** Y.S.N., A.Z.L., G.P., and M.W. have nothing to disclose. D.M.T. reports personal fees from Khondrion, Imel, Pretzel therapeutics, Reneo, and Nanna therapeutics, outside the submitted work.

## References

[1] Ng YS, Bindoff LA, Gorman GS, et al. Mitochondrial disease in adults: recent advances and future promise. Lancet Neurol. 2021;20(7):573-584. 34146515 10.1016/S1474-4422(21)00098-3

[2] Chapman J, Ng YS, Nicholls TJ. The maintenance of mitochondrial DNA integrity and dynamics by mitochondrial membranes. Life (Basel, Switzerland). 2020; 10:164. 10.3390/life10090164 PMC7555930 32858900

[3] Rath S, Sharma R, Gupta R, et al. MitoCarta3.0: an updated mitochondrial proteome now with sub-organelle localization and pathway annotations. Nucleic Acids Res. 2021;49(D1):D1541-D1547. 33174596 10.1093/nar/gkaa1011 PMC7778944

[4] Viscomi C, Zeviani M. MtDNA-maintenance defects: syndromes and genes. J Inherit Metab Dis. 2017;40(4):587-599. 28324239 10.1007/s10545-017-0027-5 PMC5500664

[5] Thompson K, Collier JJ, Glasgow RIC, et al. Recent advances in understanding the molecular genetic basis of mitochondrial disease. J Inherit Metab Dis. 2020;43(1):36-50. 31021000 10.1002/jimd.12104 PMC7041634

[6] Stenton SL, Prokisch H. Genetics of mitochondrial diseases: identifying mutations to help diagnosis. Ebiomedicine. 2020;56:102784. 32454403 10.1016/j.ebiom.2020.102784 PMC7248429

[7] Ferreira CR, Rahman S, Keller M, Zschocke J; ICIMD Advisory Group. An international classification of inherited metabolic disorders (ICIMD). J Inherit Metab Dis. 2021;44(1):164-177. 33340416 10.1002/jimd.12348 PMC9021760

[8] Taylor RW, Turnbull DM. Mitochondrial DNA mutations in human disease. Nat Rev Genet. 2005;6(5):389-402. 15861210 10.1038/nrg1606 PMC1762815

[9] Filosto M, Mancuso M, Vives-Bauza C, et al. Lack of paternal inheritance of muscle mitochondrial DNA in sporadic mitochondrial myopathies. Ann Neurol. 2003;54(4):524-526. 14520667 10.1002/ana.10709

[10] Taylor RW, McDonnell MT, Blakely EL, et al. Genotypes from patients indicate no paternal mitochondrial DNA contribution. Ann Neurol. 2003;54(4):521-524. 14520666 10.1002/ana.10673

[11] Rius R, Cowley MJ, Riley L, Puttick C, Thorburn DR, Christodoulou J. Biparental inheritance of mitochondrial DNA in humans is not a common phenomenon. Genet Med. 2019;21(12):2823-2826. 31171843 10.1038/s41436-019-0568-0

[12] Bernardino Gomes TM, Ng YS, Pickett SJ, Turnbull DM, Vincent AE. Mitochondrial DNA disorders: from pathogenic variants to preventing transmission. Hum Mol Genet. 2021;30(20):R245-R253. 34169319 10.1093/hmg/ddab156 PMC8490015

[13] Grady JP, Pickett SJ, Ng YS, et al. mtDNA heteroplasmy level and copy number indicate disease burden in m.3243A>G mitochondrial disease. EMBO Mol Med. 2018; 10:e8262. 29735722 10.15252/emmm.201708262 PMC5991564

[14] Carelli V, d’Adamo P, Valentino ML, et al. Parsing the differences in affected with LHON: genetic versus environmental triggers of disease conversion. Brain. 2016;139(Pt 3):e17. 26657166 10.1093/brain/awv339 PMC6080496

[15] Kirkman MA, Yu-Wai-Man P, Korsten A, et al. Gene-environment interactions in Leber hereditary optic neuropathy. Brain. 2009;132(Pt 9):2317-2326. 19525327 10.1093/brain/awp158 PMC2732267

[16] Gorman GS, Schaefer AM, Ng Y, et al. Prevalence of nuclear and mitochondrial DNA mutations related to adult mitochondrial disease. Ann Neurol. 2015;77(5):753-759. 25652200 10.1002/ana.24362 PMC4737121

[17] Gorman GS, Chinnery PF, DiMauro S, et al. Mitochondrial diseases. Nat Rev Dis Primers. 2016;2:16080. 27775730 10.1038/nrdp.2016.80

[18] Holt IJ, Harding AE, Morgan-Hughes JA. Deletions of muscle mitochondrial DNA in patients with mitochondrial myopathies. Nature. 1988;331(6158):717-719. 2830540 10.1038/331717a0

[19] Goto Y, Nonaka I, Horai S. A mutation in the tRNA(Leu)(UUR) gene associated with the MELAS subgroup of mitochondrial encephalomyopathies. Nature 1990; 348:651-653. 2102678 10.1038/348651a0

[20] Ballinger SW, Shoffner JM, Hedaya EV, et al. Maternally transmitted diabetes and deafness associated with a 10.4 kb mitochondrial DNA deletion. Nat Genet. 1992;1(1):11-15. 1301992 10.1038/ng0492-11

[21] van den Ouweland JM, Lemkes HH, Ruitenbeek W, et al. Mutation in mitochondrial tRNA(Leu)(UUR) gene in a large pedigree with maternally transmitted type II diabetes mellitus and deafness. Nat Genet. 1992;1(5):368-371. 1284550 10.1038/ng0892-368

[22] van den Ouweland JMW, Lemkes HHPJ, Trembath RC, et al. Maternally inherited diabetes and deafness is a distinct subtype of diabetes and associates with a single point mutation in the mitochondrial tRNA Leu(UUR) gene. Diabetes. 1994; 43:746-751. 7910800 10.2337/diab.43.6.746

[23] Maassen JA, Kadowaki T. Maternally inherited diabetes and deafness: a new subtype. Diabetologia. 1996; 39:375-382. 8777986 10.1007/BF00400668

[24] Guillausseau PJ, Massin P, Dubois-LaForgue D, et al. Maternally inherited diabetes and deafness: a multicenter study. Ann Intern Med. 2001;134(9 Pt 1):721-728. 11329229 10.7326/0003-4819-134-9_part_1-200105010-00008

[25] Whittaker RG, Schaefer AM, McFarland R, Taylor RW, Walker M, Turnbull DM. Prevalence and progression of diabetes in mitochondrial disease. Diabetologia. 2007; 50:2085-2089. 17653689 10.1007/s00125-007-0779-9

[26] Katagiri H, Asano T, Ishihara H, et al. Mitochondrial diabetes mellitus: prevalence and clinical characterization of diabetes due to mitochondrial tRNA(Leu(UUR)) gene mutation in Japanese patients. Diabetologia. 1994;37(5):504-510. 8056189 10.1007/s001250050139

[27] Pozzilli P, Pieralice S. Latent autoimmune diabetes in adults: current status and new horizons. Endocrinol Metab (Seoul). 2018;33(2):147-159. 29947172 10.3803/EnM.2018.33.2.147 PMC6021307

[28] Naylor R, Knight Johnson A, del Gaudio D. Maturity-onset diabetes of the young overview. In: Adam MP, Ardinger HH, Pagon RA, Wallace SE, Bean LJH, Mirzaa G, Amemiya A, eds. GeneReviews. Seattle (WA): University of Washington, Seattle; 1993. 29792621

[29] Riddle MC, Philipson LH, Rich SS, et al. Monogenic diabetes: from genetic insights to population-based precision in care. Reflections from a diabetes care editors’ expert forum. Diabetes Care. 2020;43(12):3117-3128. 33560999 10.2337/dci20-0065 PMC8162450

[30] Guillausseau PJ, Dubois-Laforgue D, Massin P, et al. Heterogeneity of diabetes phenotype in patients with 3243 bp mutation of mitochondrial DNA (Maternally Inherited Diabetes and Deafness or MIDD). Diabet Metab. 2004; 30:181-186. 10.1016/s1262-3636(07)70105-2 15223991

[31] Suzuki S, Hinokio Y, Hirai S, et al. Pancreatic beta-cell secretory defect associated with mitochondrial point mutation of the tRNA(LEU(UUR)) gene: a study in seven families with mitochondrial encephalomyopathy, lactic acidosis and stroke-like episodes (MELAS). Diabetologia. 1994;37(8):818-825. 7988784 10.1007/BF00404339

[32] Kadowaki H, Tobe K, Mori Y, et al. Mitochondrial gene mutation and insulin-deficient type of diabetes mellitus. Lancet. 1993;341(8849):893-894. 10.1016/0140-6736(93)93101-6 8096591

[33] Suzuki S, Oka Y, Kadowaki T, et al; Research Committee or Specific Types of Diabetes Mellitus with Gene Mutations of the Japan Diabetes Society. Clinical features of diabetes mellitus with the mitochondrial DNA 3243 (A-G) mutation in Japanese: maternal inheritance and mitochondria-related complications. Diabetes Res Clin Pract. 2003;59(3):207-217. 12590018 10.1016/s0168-8227(02)00246-2

[34] Hawa MI, Kolb H, Schloot N, et al; Action LADA consortium. Adult-onset autoimmune diabetes in Europe is prevalent with a broad clinical phenotype: action LADA 7. Diabetes Care. 2013;36(4):908-913. 23248199 10.2337/dc12-0931 PMC3609504

[35] Kobayashi T, Oka Y, Katagiri H, et al. Association between HLA and islet cell antibodies in diabetic patients with a mitochondrial DNA mutation at base pair 3243. Diabetologia. 1996;39(10):1196-1200. 8897007 10.1007/BF02658506

[36] Suzuki Y, Atsumi Y, Matsuoka K, et al. Mitochondrial tRNA(Leu(UUR)) mutation at position 3243 detected in patients with type 1 diabetes. Diabetes Res Clin Pract. 2005;67(1):92-94. 15620439 10.1016/j.diabres.2004.09.010

[37] Maassen JA . Mitochondrial diabetes: pathophysiology, clinical presentation, and genetic analysis. Am J Med Genet. 2002;115(1):66-70. 12116179 10.1002/ajmg.10346

[38] Chinnery PF, Zwijnenburg PJ, Walker M, et al. Nonrandom tissue distribution of mutant mtDNA. Am J Med Genet. 1999;85(5):498-501. 10405450

[39] McCarthy MI . Painting a new picture of personalised medicine for diabetes. Diabetologia. 2017;60(5):793-799. 28175964 10.1007/s00125-017-4210-x PMC6518376

[40] Lango Allen H, Johansson S, Ellard S, et al. Polygenic risk variants for type 2 diabetes susceptibility modify age at diagnosis in monogenic HNF1A diabetes. Diabetes. 2010;59(1):266-271. 19794065 10.2337/db09-0555 PMC2797932

[41] Murphy R, Turnbull DM, Walker M, Hattersley AT. Clinical features, diagnosis and management of maternally inherited diabetes and deafness (MIDD) associated with the 3243A>G mitochondrial point mutation. Diabet Med. 2008;25(4):383-399. 18294221 10.1111/j.1464-5491.2008.02359.x

[42] Hougaard DD, Hestoy DH, Hojland AT, Gailhede M, Petersen MB. Audiological and vestibular findings in subjects with MELAS syndrome. J Int Adv Otol. 2019;15(2):296-303. 31347509 10.5152/iao.2019.5913 PMC6750775

[43] Iwanicka-Pronicka K, Pollak A, Skórka A, et al. Postlingual hearing loss as a mitochondrial 3243A>G mutation phenotype. Plos One. 2012;7(10):e44054. 23133508 10.1371/journal.pone.0044054 PMC3485002

[44] Uimonen S, Moilanen JS, Sorri M, Hassinen IE, Majamaa K. Hearing impairment in patients with 3243A>G mtDNA mutation: phenotype and rate of progression. Hum Genet. 2001; 108:284-289. 11379873 10.1007/s004390100475

[45] Choo-Kang AT, Lynn S, Taylor GA, et al. Defining the importance of mitochondrial gene defects in maternally inherited diabetes by sequencing the entire mitochondrial genome. Diabetes. 2002;51(7):2317-2320. 12086967 10.2337/diabetes.51.7.2317

[46] Pickett SJ, Grady JP, Ng YS, et al. Phenotypic heterogeneity in m.3243A>G mitochondrial disease: the role of nuclear factors. Ann Clin Transl Neurol. 2018;5(3):333-345. 29560378 10.1002/acn3.532 PMC5846390

[47] Whittaker RG, Schaefer AM, McFarland R, Taylor RW, Walker M, Turnbull DM. Diabetes and deafness. Is it sufficient to screen for the mitochondrial 3243A>G mutation alone? Diabet Care. 2007; 30:2238-2239. 10.2337/dc07-0466 17540956

[48] Gandhi SS, Muraresku C, McCormick EM, Falk MJ, McCormack SE. Risk factors for poor bone health in primary mitochondrial disease. J Inherit Metab Dis. 2017;40(5):673-683. 28451918 10.1007/s10545-017-0046-2 PMC5659975

[49] Chow J, Rahman J, Achermann JC, Dattani MT, Rahman S. Mitochondrial disease and endocrine dysfunction. Nat Rev Endocrinol. 2017;13(2):92-104. 27716753 10.1038/nrendo.2016.151

[50] Pitceathly RD, Smith C, Fratter C, et al. Adults with RRM2B-related mitochondrial disease have distinct clinical and molecular characteristics. Brain. 2012;135(Pt 11):3392-3403. 23107649 10.1093/brain/aws231 PMC3501970

[51] Yu-Wai-Man P, Griffiths PG, Gorman GS, et al. Multi-system neurological disease is common in patients with OPA1 mutations. Brain. 2010;133(Pt 3):771-786. 20157015 10.1093/brain/awq007 PMC2842512

[52] Horvath R, Hudson G, Ferrari G, et al. Phenotypic spectrum associated with mutations of the mitochondrial polymerase gamma gene. Brain. 2006;129(Pt 7):1674-1684. 16621917 10.1093/brain/awl088

[53] Massin P, Dubois-Laforgue D, Meas T, et al; GEDIAM (Mitochondrial Diabetes French Study Group). Retinal and renal complications in patients with a mutation of mitochondrial DNA at position 3,243 (maternally inherited diabetes and deafness). A case-control study. Diabetologia. 2008;51(9):1664-1670. 18581092 10.1007/s00125-008-1073-1

[54] Iwasaki N, Babazono T, Tsuchiya K, et al. Prevalence of A-to-G mutation at nucleotide 3243 of the mitochondrial tRNA(Leu(UUR)) gene in Japanese patients with diabetes mellitus and end stage renal disease. J Hum Genet. 2001;46(6):330-334. 11393536 10.1007/s100380170068

[55] Nile DL, Brown AE, Kumaheri MA, et al. Age-related mitochondrial DNA depletion and the impact on pancreatic Beta cell function. Plos One. 2014;9(12):e115433. 25532126 10.1371/journal.pone.0115433 PMC4274008

[56] Silva JP, Köhler M, Graff C, et al. Impaired insulin secretion and beta-cell loss in tissue-specific knockout mice with mitochondrial diabetes. Nat Genet. 2000;26(3):336-340. 11062475 10.1038/81649

[57] Koeck T, Olsson AH, Nitert MD, et al. A common variant in TFB1M is associated with reduced insulin secretion and increased future risk of type 2 diabetes. Cell Metab. 2011;13(1):80-91. 21195351 10.1016/j.cmet.2010.12.007

[58] Nicholas LM, Valtat B, Medina A, et al. Mitochondrial transcription factor B2 is essential for mitochondrial and cellular function in pancreatic β-cells. Mol Metab. 2017;6(7):651-663. 28702322 10.1016/j.molmet.2017.05.005 PMC5485242

[59] Otabe S, Yasuda K, Mori Y, et al. Molecular and histological evaluation of pancreata from patients with a mitochondrial gene mutation associated with impaired insulin secretion. Biochem Biophys Res Commun. 1999;259(1):149-156. 10334931 10.1006/bbrc.1999.0650

[60] Kobayashi T, Nakanishi K, Nakase H, et al. In situ characterization of islets in diabetes with a mitochondrial DNA mutation at nucleotide position 3243. Diabetes. 1997;46(10):1567-1571. 9313751 10.2337/diacare.46.10.1567

[61] Lynn S, Borthwick GM, Charnley RM, Walker M, Turnbull DM. Heteroplasmic ratio of the A3243G mitochondrial DNA mutation in single pancreatic beta cells. Diabetologia. 2003;46(2):296-299. 12627331 10.1007/s00125-002-1018-z

[62] Brändle M, Lehmann R, Maly FE, Schmid C, Spinas GA. Diminished insulin secretory response to glucose but normal insulin and glucagon secretory responses to arginine in a family with maternally inherited diabetes and deafness caused by mitochondrial tRNA(LEU(UUR)) gene mutation. Diabetes Care. 2001;24(7):1253-1258. 11423511 10.2337/diacare.24.7.1253

[63] Velho G, Byrne MM, Clément K, et al. Clinical phenotypes, insulin secretion, and insulin sensitivity in kindreds with maternally inherited diabetes and deafness due to mitochondrial tRNALeu(UUR) gene mutation. Diabetes. 1996;45(4):478-487. 8603770 10.2337/diab.45.4.478

[64] Brown AE, Walker M. Genetics of insulin resistance and the metabolic syndrome. Curr Cardiol Rep. 2016;18(8):75. 27312935 10.1007/s11886-016-0755-4 PMC4911377

[65] Lindroos MM, Majamaa K, Tura A, et al. m.3243A>G mutation in mitochondrial DNA leads to decreased insulin sensitivity in skeletal muscle and to progressive beta-cell dysfunction. Diabetes. 2009;58(3):543-549. 19073775 10.2337/db08-0981 PMC2646052

[66] Langdahl JH, Frederiksen AL, Vissing J, Frost M, Yderstræde KB, Andersen PH. Mitochondrial mutation m.3243A>G associates with insulin resistance in non-diabetic carriers. Endocr Connect. 2019;8(7):829-837. 31146262 10.1530/EC-19-0118 PMC6590205

[67] Lindroos MM, Borra R, Mononen N, et al. Mitochondrial diabetes is associated with insulin resistance in subcutaneous adipose tissue but not with increased liver fat content. J Inherit Metab Dis. 2011;34(6):1205-1212. 21556834 10.1007/s10545-011-9338-0

[68] McKnight CL, Low YC, Elliott DA, Thorburn DR, Frazier AE. Modelling mitochondrial disease in human pluripotent stem cells: what have we learned? Int J Mol Sci. 2021; 22:7730. 34299348 10.3390/ijms22147730 PMC8306397

[69] Hämäläinen RH, Manninen T, Koivumäki H, Kislin M, Otonkoski T, Suomalainen A. Tissue- and cell-type-specific manifestations of heteroplasmic mtDNA 3243A>G mutation in human induced pluripotent stem cell-derived disease model. Proc Natl Acad Sci U S A. 2013;110(38):E3622-E3630. 24003133 10.1073/pnas.1311660110 PMC3780874

[70] Ma H, Folmes CD, Wu J, et al. Metabolic rescue in pluripotent cells from patients with mtDNA disease. Nature. 2015;524(7564):234-238. 26176921 10.1038/nature14546

[71] Yokota M, Hatakeyama H, Okabe S, Ono Y, Goto Y. Mitochondrial respiratory dysfunction caused by a heteroplasmic mitochondrial DNA mutation blocks cellular reprogramming. Hum Mol Genet. 2015;24(16):4698-4709. 26025377 10.1093/hmg/ddv201

[72] Klein Gunnewiek TM, Van Hugte EJH, Frega M, et al. m.3243A > G-induced mitochondrial dysfunction impairs human neuronal development and reduces neuronal network activity and synchronicity. Cell Rep. 2020;31(3):107538. 32320658 10.1016/j.celrep.2020.107538

[73] Boal RL, Ng YS, Pickett SJ, et al. Height as a clinical biomarker of disease burden in adult mitochondrial disease. J Clin Endocrinol Metab. 2019; 104:2057-2066. 30423112 10.1210/jc.2018-00957 PMC6469958

[74] Wedatilake Y, Brown RM, McFarland R, et al. SURF1 deficiency: a multi-centre natural history study. Orphanet J Rare Dis. 2013;8:96. 23829769 10.1186/1750-1172-8-96 PMC3706230

[75] Debray FG, Morin C, Janvier A, et al. LRPPRC mutations cause a phenotypically distinct form of Leigh syndrome with cytochrome c oxidase deficiency. J Med Genet. 2011;48(3):183-189. 21266382 10.1136/jmg.2010.081976

[76] Harvey JN, Barnett D. Endocrine dysfunction in Kearns-Sayre syndrome. Clin Endocrinol (Oxf). 1992;37(1):97-103. 1424198 10.1111/j.1365-2265.1992.tb02289.x

[77] Yamashita S, Nishino I, Nonaka I, Goto YI. Genotype and phenotype analyses in 136 patients with single large-scale mitochondrial DNA deletions. J Hum Genet. 2008;53(7):598-606. 18414780 10.1007/s10038-008-0289-8

[78] Wakefield SL, Lane M, Mitchell M. Impaired mitochondrial function in the preimplantation embryo perturbs fetal and placental development in the mouse. Biol Reprod. 2011;84(3):572-580. 21076083 10.1095/biolreprod.110.087262

[79] von Kleist-Retzow JC, Cormier-Daire V, Viot G, et al. Antenatal manifestations of mitochondrial respiratory chain deficiency. J Pediatr. 2003;143(2):208-212. 12970634 10.1067/S0022-3476(03)00130-6

[80] Tavares MV, Santos MJ, Domingues AP, et al. Antenatal manifestations of mitochondrial disorders. J Inherit Metab Dis. 2013;36(5):805-811. 23361304 10.1007/s10545-012-9567-x

[81] Feeney CL, Lim AZ, Fagan E, et al. A case-comparison study of pregnant women with mitochondrial disease - what to expect? Bjog. 2019;126(11):1380-1389. 30801962 10.1111/1471-0528.15667 PMC6767368

[82] Read JL, Whittaker RG, Miller N, et al. Prevalence and severity of voice and swallowing difficulties in mitochondrial disease. Int J Lang Commun Disord. 2012;47(1):106-111. 22268906 10.1111/j.1460-6984.2011.00072.x

[83] de Laat P, Zweers HE, Knuijt S, Smeitink JA, Wanten GJ, Janssen MC. Dysphagia, malnutrition and gastrointestinal problems in patients with mitochondrial disease caused by the m3243A> G mutation. Neth J Med. 2015;73(1):30-36. 26219939

[84] Ng YS, Feeney C, Schaefer AM, et al. Pseudo-obstruction, stroke, and mitochondrial dysfunction: a lethal combination. Ann Neurol. 2016;80(5):686-692. 27453452 10.1002/ana.24736 PMC5215534

[85] Matsuzaki M, Izumi T, Shishikura K, Suzuki H, Hirayama Y. Hypothalamic growth hormone deficiency and supplementary GH therapy in two patients with mitochondrial myopathy, encephalopathy, lactic acidosis and stroke-like episodes. Neuropediatrics. 2002;33(5):271-273. 12536371 10.1055/s-2002-36742

[86] Yorifuji T, Kawai M, Momoi T, et al. Nephropathy and growth hormone deficiency in a patient with mitochondrial tRNA(Leu(UUR)) mutation. J Med Genet. 1996;33(7):621-622. 8818955 10.1136/jmg.33.7.621 PMC1050677

[87] Berio A, Piazzi A. Multiple endocrinopathies (growth hormone deficiency, autoimmune hypothyroidism and diabetes mellitus) in Kearns-Sayre syndrome. Pediatr Med Chir. 2013;35(3):137-140. 23947115 10.4081/pmc.2013.48

[88] Cassandrini D, Savasta S, Bozzola M, et al. Mitochondrial DNA deletion in a child with mitochondrial encephalomyopathy, growth hormone deficiency, and hypoparathyroidism. J Child Neurol. 2006;21(11):983-985. 17092469 10.1177/08830738060210111001

[89] Yau EK, Chan KY, Au KM, Chow TC, Chan YW. A novel mitochondrial DNA deletion in a Chinese girl with Kearns-Sayre syndrome. Hong Kong Med J. 2009;15(5):374-377. 19801695

[90] Quade A, Zierz S, Klingmüller D. Endocrine abnormalities in mitochondrial myopathy with external ophthalmoplegia. Clin Investig. 1992;70(5):396-402. 10.1007/BF00235520 1600349

[91] Barberi S, Bozzola E, Berardinelli A, Meazza C, Bozzola M. Long-term growth hormone therapy in mitochondrial cytopathy. Horm Res. 2004;62(2):103-106. 15331853 10.1159/000080451

[92] Rocha V, Rocha D, Santos H, Sales Marques J. Growth hormone deficiency in a patient with mitochondrial disease. J Pediatr Endocrinol Metab. 2015;28(9-10):1003-1004. 25781523 10.1515/jpem-2014-0315

[93] Quintos JB, Hodax JK, Gonzales-Ellis BA, Phornphutkul C, Wajnrajch MP, Boney CM. Efficacy of growth hormone therapy in Kearns-Sayre syndrome: the KIGS experience. J Pediatr Endocrinol Metab. 2016;29(11):1319-1324. 27718492 10.1515/jpem-2016-0172

[94] Schaefer AM, Walker M, Turnbull DM, Taylor RW. Endocrine disorders in mitochondrial disease. Mol Cell Endocrinol. 2013;379(1-2):2-11. 23769710 10.1016/j.mce.2013.06.004 PMC3820028

[95] Isotani H, Fukumoto Y, Kawamura H, et al. Hypoparathyroidism and insulin-dependent diabetes mellitus in a patient with Kearns-Sayre syndrome harbouring a mitochondrial DNA deletion. Clin Endocrinol (Oxf). 1996;45(5):637-641. 8977763 10.1046/j.1365-2265.1996.00856.x

[96] Horwitz SJ, Roessmann U. Kearns-Sayre syndrome with hypoparathyroidism. Ann Neurol. 1978;3(6):513-518. 677816 10.1002/ana.410030611

[97] Lee YS, Yap HK, Barshop BA, Lee YS, Rajalingam S, Loke KY. Mitochondrial tubulopathy: the many faces of mitochondrial disorders. Pediatr Nephrol. 2001;16(9):710-712. 11511982 10.1007/s004670100637

[98] Sabella-Jiménez V, Otero-Herrera C, Silvera-Redondo C, Garavito-Galofre P. Mitochondrial DNA deletion and duplication in Kearns-Sayre Syndrome (KSS) with initial presentation as Pearson Marrow-Pancreas Syndrome (PMPS): two case reports in Barranquilla, Colombia. Mol Genet Genomic Med. 2020;8(11):e1509. 33030289 10.1002/mgg3.1509 PMC7667363

[99] Barca E, Long Y, Cooley V, et al. Mitochondrial diseases in North America: an analysis of the NAMDC Registry. Neurol Genet. 2020;6(2):e402. 32337332 10.1212/NXG.0000000000000402 PMC7164977

[100] Calderwood L, Holm IA, Teot LA, Anselm I. Adrenal insufficiency in mitochondrial disease: a rare case of GFER-related mitochondrial encephalomyopathy and review of the literature. J Child Neurol. 2016;31(2):190-194. 26018198 10.1177/0883073815587327

[101] Nicolino M, Ferlin T, Forest M, et al. Identification of a large-scale mitochondrial deoxyribonucleic acid deletion in endocrinopathies and deafness: report of two unrelated cases with diabetes mellitus and adrenal insufficiency, respectively. J Clin Endocrinol Metab. 1997;82(9):3063-3067. 9284744 10.1210/jcem.82.9.4220

[102] Sasano N, Tamura T, Azami T, Sasano H. Severe hyponatremia occurring after surgical stress in a patient with mitochondrial disease. J Anesth. 2009;23(4):587-590. 19921372 10.1007/s00540-009-0808-6

[103] Gorman GS, Grady JP, Turnbull DM. Mitochondrial donation–how many women could benefit? N Engl J Med. 2015;372(9):885-887. 10.1056/NEJMc1500960 PMC4481295 25629662

[104] Kang YX, Wang YJ, Zhang Q, Pang XH, Gu W. A case of hypopituitarism accompanying Kearns-Sayre syndrome treated with human chorionic gonadotropin: a case report and literature review. Andrologia. 2017;49: e12711. 10.1111/and.12711 27709644

[105] Ding Y, Xia BH, Zhuo GC, Zhang CJ, Leng JH. Premature ovarian insufficiency may be associated with the mutations in mitochondrial tRNA genes. Endocr J. 2019;66(1):81-88. 30404982 10.1507/endocrj.EJ18-0308

[106] Carod-Artal FJ, Herrero MD, Lara MC, et al. Cognitive dysfunction and hypogonadotrophic hypogonadism in a Brazilian patient with mitochondrial neurogastrointestinal encephalomyopathy and a novel ECGF1 mutation. Eur J Neurol. 2007;14(5):581-585. 17437622 10.1111/j.1468-1331.2007.01720.x

[107] Kalkan IH, Tayfur O, Oztaş E, Beyazit Y, Yildiz H, Tunç B. A novel finding in MNGIE (mitochondrial neurogastrointestinal encephalomyopathy): hypergonadotropic hypogonadism. Hormones (Athens). 2012;11(3):377-379. 22908072 10.14310/horm.2002.1368

[108] Lönnqvist T, Paetau A, Valanne L, Pihko H. Recessive twinkle mutations cause severe epileptic encephalopathy. Brain. 2009;132(Pt 6):1553-1562. 19304794 10.1093/brain/awp045

[109] Dallabona C, Diodato D, Kevelam SH, et al. Novel (ovario) leukodystrophy related to AARS2 mutations. Neurology. 2014; 82:2063-2071. 24808023 10.1212/WNL.0000000000000497 PMC4118500

[110] Pagnamenta AT, Taanman JW, Wilson CJ, et al. Dominant inheritance of premature ovarian failure associated with mutant mitochondrial DNA polymerase gamma. Hum Reprod. 2006;21(10):2467-2473. 16595552 10.1093/humrep/del076

[111] Tiosano D, Mears JA, Buchner DA. Mitochondrial dysfunction in primary ovarian insufficiency. Endocrinology. 2019;160(10):2353-2366. 31393557 10.1210/en.2019-00441 PMC6760336

[112] Filosto M, Mancuso M, Nishigaki Y, et al. Clinical and genetic heterogeneity in progressive external ophthalmoplegia due to mutations in polymerase gamma. Arch Neurol. 2003;60(9):1279-1284. 12975295 10.1001/archneur.60.9.1279

[113] Newman WG, Friedman TB, Conway GS, Demain LAM. Perrault syndrome. In: Adam MP, Ardinger HH, Pagon RA, Wallace SE, Bean LJH, Stephens K, Amemiya A, eds. GeneReviews. Seattle (WA): University of Washington, Seattle; 1993. 25254289

[114] Demain LA, Urquhart JE, O’Sullivan J, et al. Expanding the genotypic spectrum of Perrault syndrome. Clin Genet. 2017;91(2):302-312. 26970254 10.1111/cge.12776

[115] Jenkinson EM, Rehman AU, Walsh T, et al; University of Washington Center for Mendelian Genomics. Perrault syndrome is caused by recessive mutations in CLPP, encoding a mitochondrial ATP-dependent chambered protease. Am J Hum Genet. 2013;92(4):605-613. 23541340 10.1016/j.ajhg.2013.02.013 PMC3617381

[116] Pierce SB, Gersak K, Michaelson-Cohen R, et al. Mutations in LARS2, encoding mitochondrial leucyl-tRNA synthetase, lead to premature ovarian failure and hearing loss in Perrault syndrome. Am J Hum Genet. 2013;92(4):614-620. 23541342 10.1016/j.ajhg.2013.03.007 PMC3617377

[117] Pierce SB, Chisholm KM, Lynch ED, et al. Mutations in mitochondrial histidyl tRNA synthetase HARS2 cause ovarian dysgenesis and sensorineural hearing loss of Perrault syndrome. Proc Natl Acad Sci U S A. 2011;108(16):6543-6548. 21464306 10.1073/pnas.1103471108 PMC3081023

[118] Chatzispyrou IA, Alders M, Guerrero-Castillo S, et al. A homozygous missense mutation in ERAL1, encoding a mitochondrial rRNA chaperone, causes Perrault syndrome. Hum Mol Genet. 2017;26(13):2541-2550. 28449065 10.1093/hmg/ddx152 PMC5965403

[119] Pierce SB, Walsh T, Chisholm KM, et al. Mutations in the DBP-deficiency protein HSD17B4 cause ovarian dysgenesis, hearing loss, and ataxia of Perrault syndrome. Am J Hum Genet. 2010;87(2):282-288. 20673864 10.1016/j.ajhg.2010.07.007 PMC2917704

[120] Marlin S, Lacombe D, Jonard L, et al. Perrault syndrome: report of four new cases, review and exclusion of candidate genes. Am J Med Genet A. 2008;146A(5):661-664. 18241061 10.1002/ajmg.a.32180

[121] Lerat J, Jonard L, Loundon N, et al. An application of NGS for molecular investigations in Perrault syndrome: study of 14 families and review of the literature. Hum Mutat. 2016;37(12):1354-1362. 27650058 10.1002/humu.23120

[122] van der Knaap MS, Bugiani M, Mendes MI, et al. Biallelic variants in LARS2 and KARS cause deafness and (ovario)leukodystrophy. Neurology. 2019;92(11):e1225-e1237. 30737337 10.1212/WNL.0000000000007098 PMC9281382

[123] Say RE, Whittaker RG, Turnbull HE, McFarland R, Taylor RW, Turnbull DM. Mitochondrial disease in pregnancy: a systematic review. Obstet Med. 2011;4(3):90-94. 27579099 10.1258/om.2011.110008 PMC4989604

[124] Yu-Wai-Man P, Newman NJ, Carelli V, et al. Bilateral visual improvement with unilateral gene therapy injection for Leber hereditary optic neuropathy. Sci Transl Med. 2020; 12(573):eaaz7423. 33298565 10.1126/scitranslmed.aaz7423

[125] National Institute for Health and Care Excellence (NICE). Guidelines - diabetes in pregnancy: management from preconception to the postnatal period. https://www.nice.org.uk/guidance/ng3. Accessed May 10, 2021. 32212588

[126] Hikmat O, Naess K, Engvall M, et al. The impact of gender, puberty, and pregnancy in patients with POLG disease. Ann Clin Transl Neurol. 2020;7(10):2019-2025. 32949115 10.1002/acn3.51199 PMC7545595

[127] Orsucci D, Caldarazzo Ienco E, Rossi A, Siciliano G, Mancuso M. Mitochondrial syndromes revisited. J Clin Med. 2021; 10:1249. 33802970 10.3390/jcm10061249 PMC8002645

[128] Zia N, Nikookam Y, Muzaffar J, Kullar P, Monksfield P, Bance M. Cochlear implantation outcomes in patients with mitochondrial hearing loss: a systematic review and narrative synthesis. J Int Adv Otol. 2021;17(1):72-80. 33605225 10.5152/iao.2020.9226 PMC7901420

[129] Ng YS, Bindoff LA, Gorman GS, et al. Consensus-based statements for the management of mitochondrial stroke-like episodes. Wellcome Open Res. 2019;4:201. 32090171 10.12688/wellcomeopenres.15599.1 PMC7014928

[130] Chu BC, Terae S, Takahashi C, et al. MRI of the brain in the Kearns-Sayre syndrome: report of four cases and a review. Neuroradiology. 1999;41(10):759-764. 10552027 10.1007/s002340050838

[131] Quijada-Fraile P, O’Callaghan M, Martín-Hernández E, et al. Follow-up of folinic acid supplementation for patients with cerebral folate deficiency and Kearns-Sayre syndrome. Orphanet J Rare Dis. 2014;9:217. 25539952 10.1186/s13023-014-0217-2 PMC4302586

[132] Stefanetti RJ, Blain A, Jimenez-Moreno C, et al. Measuring the effects of exercise in neuromuscular disorders: a systematic review and meta-analyses. Wellcome Open Res. 2020;5:84. 32671231 10.12688/wellcomeopenres.15825.1 PMC7331112

[133] de Laat P, Smeitink JAM, Janssen MCH, Keunen JEE, Boon CJF. Mitochondrial retinal dystrophy associated with the m.3243A>G mutation. Ophthalmology. 2013;120(12):2684-2696. 23806424 10.1016/j.ophtha.2013.05.013

[134] Bates MG, Bourke JP, Giordano C, d’Amati G, Turnbull DM, Taylor RW. Cardiac involvement in mitochondrial DNA disease: clinical spectrum, diagnosis, and management. Eur Heart J. 2012;33(24):3023-3033. 22936362 10.1093/eurheartj/ehs275 PMC3530901

[135] Wellcome Centre for Mitochondrial Research, UK. https://www.newcastle-mitochondria.com/wp-content/cache/all/guidelines/index.html. Accessed May 27, 2021.

[136] DiMauro S, Schon EA, Carelli V, Hirano M. The clinical maze of mitochondrial neurology. Nat Rev Neurol. 2013;9(8):429-444. 23835535 10.1038/nrneurol.2013.126 PMC3959773

[137] Quadir A, Pontifex CS, Lee Robertson H, Labos C, Pfeffer G. Systematic review and meta-analysis of cardiac involvement in mitochondrial myopathy. Neurol Genet. 2019;5(4):e339. 31403078 10.1212/NXG.0000000000000339 PMC6659349

[138] Wahbi K, Bougouin W, Béhin A, et al. Long-term cardiac prognosis and risk stratification in 260 adults presenting with mitochondrial diseases. Eur Heart J. 2015;36(42):2886-2893. 26224072 10.1093/eurheartj/ehv307

[139] Haghighi A, Haack TB, Atiq M, et al. Sengers syndrome: six novel AGK mutations in seven new families and review of the phenotypic and mutational spectrum of 29 patients. Orphanet J Rare Dis. 2014; 9:1-12. 25208612 10.1186/s13023-014-0119-3 PMC4167147

[140] Taylor RW, Giordano C, Davidson MM, et al. A homoplasmic mitochondrial transfer ribonucleic acid mutation as a cause of maternally inherited hypertrophic cardiomyopathy. J Am Coll Cardiol. 2003;41(10):1786-1796. 12767666 10.1016/s0735-1097(03)00300-0

[141] Papadopoulos C, Wahbi K, Behin A, et al. Incidence and predictors of total mortality in 267 adults presenting with mitochondrial diseases. J Inherit Metab Dis. 2020;43(3):459-466. 31652339 10.1002/jimd.12185

[142] Malfatti E, Laforêt P, Jardel C, et al. High risk of severe cardiac adverse events in patients with mitochondrial m.3243A>G mutation. Neurology. 2013;80(1):100-105. 23243073 10.1212/WNL.0b013e31827b1a2f

[143] Ng YS, Grady JP, Lax NZ, et al. Sudden adult death syndrome in m.3243A>G-related mitochondrial disease: an unrecognized clinical entity in young, asymptomatic adults. Eur Heart J. 2016;37(32):2552-2559. 26188002 10.1093/eurheartj/ehv306 PMC5008417

[144] Barends M, Verschuren L, Morava E, Nesbitt V, Turnbull D, McFarland R. Causes of death in adults with mitochondrial disease. JIMD Rep. 2016;26:103-113. 26354038 10.1007/8904_2015_449 PMC4864865

[145] Lim AZ, Jones DM, Bates MGD, et al. Risk of cardiac manifestations in adult mitochondrial disease caused by nuclear genetic defects. Open Heart. 2021; 8:e001510.

[146] Amiot A, Tchikviladzé M, Joly F, et al. Frequency of mitochondrial defects in patients with chronic intestinal pseudo-obstruction. Gastroenterology. 2009;137(1):101-109. 19344718 10.1053/j.gastro.2009.03.054

[147] Hirano M, Silvestri G, Blake DM, et al. Mitochondrial neurogastrointestinal encephalomyopathy (MNGIE): clinical, biochemical, and genetic features of an autosomal recessive mitochondrial disorder. Neurology. 1994;44(4):721-727. 8164833 10.1212/wnl.44.4.721

[148] Nishino I, Spinazzola A, Hirano M. Thymidine phosphorylase gene mutations in MNGIE, a human mitochondrial disorder. Science. 1999;283(5402):689-692. 9924029 10.1126/science.283.5402.689

[149] Shimura M, Kuranobu N, Ogawa-Tominaga M, et al. Clinical and molecular basis of hepatocerebral mitochondrial DNA depletion syndrome in Japan: evaluation of outcomes after liver transplantation. Orphanet J Rare Dis. 2020;15(1):169. 32703289 10.1186/s13023-020-01441-5 PMC7379809

[150] Parikh S, Karaa A, Goldstein A, et al. Solid organ transplantation in primary mitochondrial disease: proceed with caution. Mol Genet Metab. 2016;118(3):178-184. 27312126 10.1016/j.ymgme.2016.04.009

[151] Delarue A, Paut O, Guys JM, et al. Inappropriate liver transplantation in a child with Alpers-Huttenlocher syndrome misdiagnosed as valproate-induced acute liver failure. Pediatr Transplant. 2000;4(1):67-71. 10731063 10.1034/j.1399-3046.2000.00090.x

[152] O’Toole JF . Renal manifestations of genetic mitochondrial disease. Int J Nephrol Renovascular Dis. 2014; 7:57-67. 10.2147/IJNRD.S37887 PMC3916636 24516335

[153] Ng YS, Alston CL, Diodato D, et al. The clinical, biochemical and genetic features associated with RMND1-related mitochondrial disease. J Med Genet. 2016;53(11):768-775. 27412952 10.1136/jmedgenet-2016-103910 PMC5264221

[154] Seidowsky A, Hoffmann M, Glowacki F, et al. Renal involvement in MELAS syndrome - a series of 5 cases and review of the literature. Clin Nephrol. 2013;80(6):456-463. 22909780 10.5414/CN107063

[155] de Laat P, van Engelen N, Wetzels JF, Smeitink JAM, Janssen MCH. Five non-mitochondrial myopathy, encephalopathy, lactic acidosis and stroke-like episodes phenotype adult patients with m.3243A>G mutation after kidney transplantation: follow-up and review of the literature. Clin Kidney J. 2019;12(6):840-846. 31807297 10.1093/ckj/sfz020 PMC6885678

[156] Musumeci O, Barca E, Lamperti C, et al. Lipomatosis incidence and characteristics in an Italian Cohort of Mitochondrial Patients. Front Neurol. 2019;10:160. 30873109 10.3389/fneur.2019.00160 PMC6402385

[157] Capel E, Vatier C, Cervera P, et al. MFN2-associated lipomatosis: clinical spectrum and impact on adipose tissue. J Clin Lipidol. 2018;12(6):1420-1435. 30158064 10.1016/j.jacl.2018.07.009

[158] Tesarova M, Vondrackova A, Stufkova H, et al. Sideroblastic anemia associated with multisystem mitochondrial disorders. Pediatr Blood Cancer. 2019;66(4):e27591. 30588737 10.1002/pbc.27591

[159] Sommerville EW, Ng YS, Alston CL, et al. Clinical features, molecular heterogeneity, and prognostic implications in YARS2-related mitochondrial myopathy. JAMA Neurol. 2017;74(6):686-694. 28395030 10.1001/jamaneurol.2016.4357 PMC5822212

[160] Suomalainen A, Elo JM, Pietiläinen KH, et al. FGF-21 as a biomarker for muscle-manifesting mitochondrial respiratory chain deficiencies: a diagnostic study. Lancet Neurol. 2011;10(9):806-818. 21820356 10.1016/S1474-4422(11)70155-7 PMC7568343

[161] Yatsuga S, Fujita Y, Ishii A, et al. Growth differentiation factor 15 as a useful biomarker for mitochondrial disorders. Ann Neurol. 2015;78(5):814-823. 26463265 10.1002/ana.24506 PMC5057301

[162] Alston CL, Stenton SL, Hudson G, Prokisch H, Taylor RW. The genetics of mitochondrial disease: dissecting mitochondrial pathology using multi-omic pipelines. J Pathol. 2021;254(4):430-442. 33586140 10.1002/path.5641 PMC8600955

[163] Rahman J, Rahman S. Mitochondrial medicine in the omics era. Lancet. 2018;391(10139):2560-2574. 29903433 10.1016/S0140-6736(18)30727-X

[164] Palculict ME, Zhang VW, Wong LJ, Wang J. Comprehensive mitochondrial genome analysis by massively parallel sequencing. Methods Mol Biol. 2016;1351:3-17. 26530670 10.1007/978-1-4939-3040-1_1

[165] Wagner M, Berutti R, Lorenz-Depiereux B, et al. Mitochondrial DNA mutation analysis from exome sequencing–a more holistic approach in diagnostics of suspected mitochondrial disease. J Inherit Metab Dis. 2019;42(5):909-917. 31059585 10.1002/jimd.12109

[166] Whittaker RG, Blackwood JK, Alston CL, et al. Urine heteroplasmy is the best predictor of clinical outcome in the m.3243A>G mtDNA mutation. Neurology. 2009;72(6):568-569. 19204268 10.1212/01.wnl.0000342121.91336.4d PMC2818183

[167] Hardy SA, Blakely EL, Purvis AI, et al. Pathogenic mtDNA mutations causing mitochondrial myopathy: the need for muscle biopsy. Neurol. Genet. 2016; 2:e82. 27536729 10.1212/NXG.0000000000000082 PMC4972142

[168] NHS Highly Specialised Service for Rare Mitochondrial Disorders, UK. https://mitochondrialdisease.nhs.uk/. Accessed May 27, 2021.

[169] Mitochondrial Care Network, USA. https://www.mitonetwork.org/. Accessed May 27, 2021.

[170] European Reference Network of Neuromuscular Diseases. https://ern-euro-nmd.eu/hcps/. Accessed May 27, 2021.

[171] Parikh S, Goldstein A, Karaa A, et al. Patient care standards for primary mitochondrial disease: a consensus statement from the Mitochondrial Medicine Society. Genet Med. 2017;19: 1380. 10.1038/gim.2017.107 PMC7804217 28749475

[172] Yeung RO, Al Jundi M, Gubbi S, et al. Management of mitochondrial diabetes in the era of novel therapies. J Diabetes Complicat. 2021;35(1):107584. 10.1016/j.jdiacomp.2020.107584 PMC7554068 32331977

[173] Bonora BM, Avogaro A, Fadini GP. Extraglycemic effects of SGLT2 inhibitors: a review of the evidence. Diabetes Metab Syndr Obes. 2020;13:161-174. 32021362 10.2147/DMSO.S233538 PMC6982447

[174] Barcelos I, Shadiack E, Ganetzky RD, Falk MJ. Mitochondrial medicine therapies: rationale, evidence, and dosing guidelines. Curr Opin Pediatr. 2020;32(6):707-718. 33105273 10.1097/MOP.0000000000000954 PMC7774245

[175] Karaa A, Kriger J, Grier J, et al. Mitochondrial disease patients’ perception of dietary supplements’ use. Mol Genet Metab. 2016;119(1-2):100-108. 27444792 10.1016/j.ymgme.2016.07.005 PMC5031526

[176] Weissig V . Drug development for the therapy of mitochondrial diseases. Trends Mol Med. 2020;26(1):40-57. 31727544 10.1016/j.molmed.2019.09.002

[177] Mandia D, Shor N, Benoist JF, Nadjar Y. Adolescent-onset and adult-onset vitamin-responsive neurogenetic diseases: a review. JAMA Neurol. 2021;78(4):483-490. 33427863 10.1001/jamaneurol.2020.4911

[178] Distelmaier F, Haack TB, Wortmann SB, Mayr JA, Prokisch H. Treatable mitochondrial diseases: cofactor metabolism and beyond. Brain. 2017;140(2):e11. 27993888 10.1093/brain/aww303

[179] Russell OM, Gorman GS, Lightowlers RN, Turnbull DM. Mitochondrial diseases: hope for the future. Cell. 2020;181(1):168-188. 32220313 10.1016/j.cell.2020.02.051

[180] Viscomi C, Zeviani M. Strategies for fighting mitochondrial diseases. J Intern Med. 2020;287(6):665-684. 32100338 10.1111/joim.13046

[181] Pitceathly RDS, Keshavan N, Rahman J, Rahman S. Moving towards clinical trials for mitochondrial diseases. J Inherit Metab Dis. 2021;44(1):22-41. 32618366 10.1002/jimd.12281 PMC8432143

[182] Klopstock T, Yu-Wai-Man P, Dimitriadis K, et al. A randomized placebo-controlled trial of idebenone in Leber’s hereditary optic neuropathy. Brain. 2011;134(Pt 9):2677-2686. 21788663 10.1093/brain/awr170 PMC3170530

[183] Yu-Wai-Man P, Newman NJ, Carelli V, et al. Bilateral visual improvement with unilateral gene therapy injection for Leber hereditary optic neuropathy. Sci Transl Med. 2020; 12:eaaz7423. 33298565 10.1126/scitranslmed.aaz7423

[184] Pitceathly RDS, Keshavan N, Rahman J, Rahman S. Moving towards clinical trials for mitochondrial diseases. J Inherit Metab Dis. 2021;44(1):22-41. 32618366 10.1002/jimd.12281 PMC8432143

[185] Bax BE . Mitochondrial neurogastrointestinal encephalomyopathy: approaches to diagnosis and treatment. J Transl Genet Genom. 2020;4:1-16. 32914088 10.20517/jtgg.2020.08 PMC7116056

[186] Garone C, Taylor RW, Nascimento A, et al. Retrospective natural history of thymidine kinase 2 deficiency. J Med Genet. 2018;55(8):515-521. 29602790 10.1136/jmedgenet-2017-105012 PMC6073909

[187] Domínguez-González C, Madruga-Garrido M, Mavillard F, et al. Deoxynucleoside therapy for thymidine kinase 2-deficient myopathy. Ann Neurol. 2019;86(2):293-303. 31125140 10.1002/ana.25506 PMC7586249

[188] Bacman SR, Kauppila JHK, Pereira CV, et al. MitoTALEN reduces mutant mtDNA load and restores tRNAAla levels in a mouse model of heteroplasmic mtDNA mutation. Nat Med. 2018;24(11):1696-1700. 30250143 10.1038/s41591-018-0166-8 PMC6942693

[189] Gammage PA, Viscomi C, Simard ML, et al. Genome editing in mitochondria corrects a pathogenic mtDNA mutation in vivo. Nat Med. 2018;24(11):1691-1695. 30250142 10.1038/s41591-018-0165-9 PMC6225988

[190] Chinnery PF, DiMauro S, Shanske S, et al. Risk of developing a mitochondrial DNA deletion disorder. Lancet. 2004;364(9434):592-596. 15313359 10.1016/S0140-6736(04)16851-7

[191] Ng YS, Martikainen MH, Gorman GS, et al. Pathogenic variants in MT-ATP6: a United Kingdom-based mitochondrial disease cohort study. Ann Neurol. 2019;86(2):310-315. 31187502 10.1002/ana.25525 PMC6771528

[192] Wilson IJ, Carling PJ, Alston CL, et al. Mitochondrial DNA sequence characteristics modulate the size of the genetic bottleneck. Hum Mol Genet. 2016;25(5):1031-1041. 26740552 10.1093/hmg/ddv626 PMC4754047

[193] Nesbitt V, Alston CL, Blakely EL, et al. A national perspective on prenatal testing for mitochondrial disease. Eur J Hum Genet. 2014;22(11):1255-1259. 24642831 10.1038/ejhg.2014.35 PMC4200441

[194] Mujezinovic F, Alfirevic Z. Procedure-related complications of amniocentesis and chorionic villous sampling: a systematic review. Obstet Gynecol. 2007;110(3):687-694. 17766619 10.1097/01.AOG.0000278820.54029.e3

[195] Poulton J, Steffann J, Burgstaller J, McFarland R; workshop participants. 243^rd^ ENMC international workshop: Developing guidelines for management of reproductive options for families with maternally inherited mtDNA disease, Amsterdam, the Netherlands, 22-24 March 2019. Neuromuscul Disord. 2019;29(9):725-733. 31501000 10.1016/j.nmd.2019.08.004

[196] Pickett SJ, Blain A, Ng YS, et al. Mitochondrial donation - which women could benefit? N Engl J Med. 2019; 380:1971-1972. 31091381 10.1056/NEJMc1808565

[197] Richardson J, Irving L, Hyslop LA, et al. Concise reviews: assisted reproductive technologies to prevent transmission of mitochondrial DNA disease. Stem Cells. 2015;33(3):639-645. 25377180 10.1002/stem.1887 PMC4359624

[198] Tachibana M, Amato P, Sparman M, et al. Towards germline gene therapy of inherited mitochondrial diseases. Nature. 2013;493(7434):627-631. 23103867 10.1038/nature11647 PMC3561483

[199] Craven L, Tuppen HA, Greggains GD, et al. Pronuclear transfer in human embryos to prevent transmission of mitochondrial DNA disease. Nature. 2010;465(7294):82-85. 20393463 10.1038/nature08958 PMC2875160

[200] Gorman GS, McFarland R, Stewart J, Feeney C, Turnbull DM. Mitochondrial donation: from test tube to clinic. Lancet. 2018;392(10154):1191-1192. 30319102 10.1016/S0140-6736(18)31868-3

[201] National Academy of Medicine, National Academy of Sciences, and the Royal Society. Heritable Human Genome Editing. Washington, DC: The National Academies Press;2020. 32897669

[202] Craven L, Herbert M, Murdoch A, Murphy J, Lawford Davies J, Turnbull DM. Research into policy: a brief history of mitochondrial donation. Stem Cells. 2016;34(2):265-267. 26418557 10.1002/stem.2221 PMC4855617

